# 4D-Printed Temperature-Switchable Monolithic Packing for NIR-Actuated Elemental Speciation Analysis

**DOI:** 10.1021/acs.analchem.6c01832

**Published:** 2026-08-26

**Authors:** Yi-Ting Chen, Yi-Chia Ho, Cheng-Kuan Su

**Affiliations:** Department of Chemistry, 34916 National Chung Hsing University, Taichung City 40227, Taiwan, R.O.C.

## Abstract

Four-dimensional printing (4DP) technologies drive the use of stimuli-responsive materials in the fabrication of versatile analytical devices. In this study, a monolithic packing comprising a nonresponsive part and a graphene-incorporated thermoresponsive part was fabricated using digital light processing 4DP technique and *N*-isopropylacrylamide (NIPAM)-based photocurable resins with and without graphene to enable elemental speciation analysis in one solid-phase extraction column. The nonresponsive part selectively extracted Cr­(III), As­(III), and Se­(IV) when its temperature was lower than the volume phase transition temperature (VPTT) of the polyNIPAM hydrogel. The graphene-incorporated part became hydrophobic for the selective extraction of Cr­(VI), As­(V), and Se­(VI) when near-infrared (NIR) irradiation was applied to increase its temperature (>VPTT), resulting in the NIR-actuated switching of monolithic packing’s hydrophobicity for the speciation analysis of inorganic Cr, As, and Se. After optimizing the design, fabrication, and NIR actuation of the monolithic packing, the automated speciation scheme was coupled with inductively coupled plasma mass spectrometry to enable highly sensitive speciation of inorganic Cr, As, and Se; the method detection limits and absolute extraction efficiencies of these species were 0.1–0.6 ng L^–1^ and >87.3%, respectively. These species were analyzed in reference materials (CASS-6, SLRS-5, 1643f, and Seronorm Trace Elements Urine L-2) and environmental water and human urine samples to validate the reliability of this method. The results indicated that the 4D-printed NIR-actuated temperature-switchable monolithic packing showed promising capability and applicability to advance nonchromatographic speciation analysis of trace elements.

## Graphical abstract

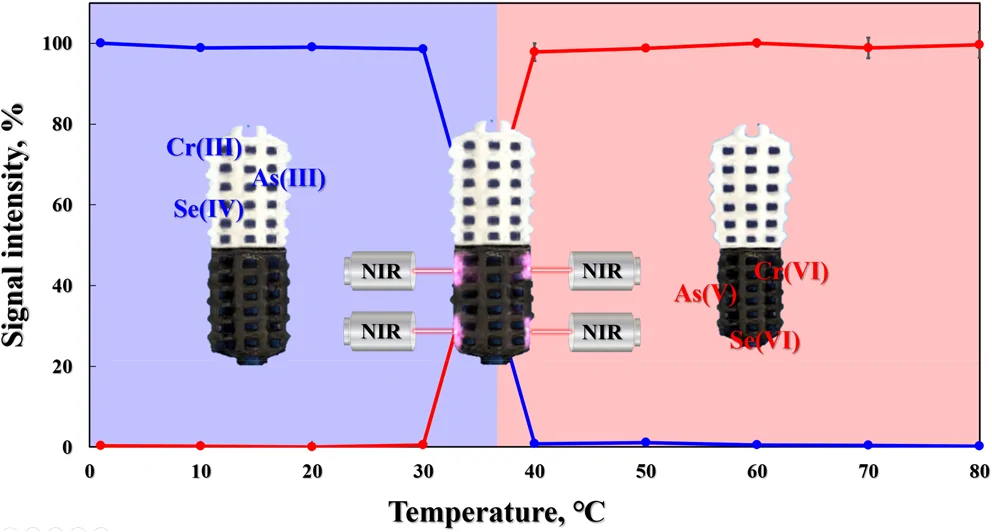

The identification and quantification of specific elemental species in environmental and biological media determine the mobility, bioavailability, and toxicity of elements and are consequently considered essential.^1–5^ Hyphenating chromatographic techniques with inductively coupled plasma mass spectrometry (ICP-MS) has established this approach as the most widely used tool for probing these elemental species in complex real samples.^4,6–8^ Nevertheless, not only analyte dilution caused by elution with mobile phases but also fluctuations in retention time and sensitivity due to the continuous introduction of salty or organic mobile phases into ICP-MS systems may generate results with poor reliability.^6–8^ Alternatively, nonchromatographic schemes capable of selectively separating elemental species have increasingly been developed and coupled with atomic spectrometric determination to achieve more reliable elemental speciation analysis.^9–12^

Among nonchromatographic speciation methods,^13–21^ solid-phase extraction (SPE)-based schemes targeting selective interactions between elemental species and sorbents have been employed to improve the analytical performance and applicability of elemental speciation analysis. These schemes typically involve at least two columns packed with either the same or two different types of sorbents and operated under different conditions to extract the corresponding species^13–17^ or one type of sorbent to extract certain species using the first column, followed by oxidation/reduction of the other species for extraction using the second column.^18–21^ Theoretically, the use of one column packed with an appropriate type of sorbent to extract all target elemental species for elution and determination may enhance the applicability of speciation schemes for routine applications. Moreover, when coupled with atomic spectrometric methods, the automation of SPE-based elemental speciation schemes using a closed analytical system substantially minimizes blank contributions, species transformation due to environmental redox reactions, and errors from manual operation.^9,11,12,21–23^ However, devising more efficient and versatile sorbents that can advance SPE-based elemental speciation schemes and facilitate their automation remains a significant challenge.^24,25^

Owing to their stimuli-responsive properties and extraction capacities, stimuli-responsive hydrogels have recently been used as effective sorbents for extracting analytes of interest.^26–29^ Tokuyama and Iwama prepared a poly­(*N*-isopropylacrylamide) (polyNIPAM) hydrogel for adsorption of Cu­(II)-di-n-dodecylbenzenesulfonate complexes through a hydrophobic interaction by adjusting the solution temperature to be higher than the volume phase transition temperature (VPTT); subsequently, these complexes were desorbed from the hydrogel by adjusting the temperature to be lower than the VPTT.^30^ Chen et al. prepared a polyNIPAM–magnetite composite hydrogel with 3-(trimethoxysilyl)­propyl methacrylate as the bridging agent for enhanced Cr­(III) adsorption by increasing the solution temperature from 25 to 50 °C (>VPTT), followed by desorption when the solution was cooled back.^31^ Zhang et al. prepared a cellulose filament-reinforced poly­(NIPAM-*co*-acrylic acid) hydrogel for pH- and temperature-responsive adsorption (pH 6.0 and 20 °C) and desorption (pH 3.0 and 60 °C) of Pb­(II).^32^ Nonetheless, these stimuli-responsive hydrogels are usually synthesized in a random shape,^30–35^ and preparing stimuli-responsive hydrogels with functional geometric features may be technically difficult and time-consuming. More effective fabrication strategies that enable stimuli-responsive hydrogels with tunable geometric functions and actuatable switching of their physicochemical properties can provide larger surface areas and higher extraction efficiencies, thereby advancing their use in the development of SPE-based elemental speciation methods.

As an alternative to conventional template-assisted fabrication methods, three-dimensional printing (3DP) technologies have reshaped the geometric functions, adaptability, and analytical performance of hydrogels.^36–39^ Four-dimensional printing (4DP) technologies employing conventional 3DP techniques with stimuli-responsive materials are enabling stimuli-responsive hydrogels to possess stimuli-induced time-dependent switching of their physicochemical properties as the fourth dimension of printed devices.^40–43^ Despite the use of 4D-printed hydrogels in drug delivery and biomedical applications,^44–46^ analytical methods focusing on the desirable stimuli-responsive properties of 4D-printed hydrogels are rare.^26,47^ Furthermore, 4D-printed hydrogels have not yet been utilized in the development of automated elemental speciation schemes.^48^ The use of *in situ* stimuli-activated switching of the physicochemical properties of 4D-printed hydrogels to selectively extract and/or elute target elemental species and thereby advance the applicability of SPE-based elemental speciation schemes and automated speciation systems is promising.

In the present study, a monolithic packing comprising a nonresponsive part and a near-infrared (NIR)-actuated graphene-incorporated part was fabricated using digital light processing (DLP) 4DP technique and NIPAM-based photocurable resins with and without graphene to achieve the goal of using one SPE column to develop an automated system for the speciation analysis of inorganic Cr, As, and Se. The nonresponsive part selectively extracted Cr­(III), As­(III), and Se­(IV) when its temperature was lower than the VPTT of the polyNIPAM hydrogel. The graphene-incorporated part became hydrophobic for the selective extraction of Cr­(VI), As­(V), and Se­(VI) when NIR irradiation was applied to increase its temperature (>VPTT). Dissimilar to conventional SPE-based elemental speciation schemes that use two columns or require prereduction or preoxidation of target elemental species,^13–21^ thermoresponsive hydrogel and NIR actuation were combined to realize the *in situ* switching of the hydrophobicity of the monolithic packing for the selective extraction and desorption of elemental species in only one SPE column, achieving a simpler speciation scheme coupled with ICP-MS for a reliable speciation analysis of inorganic Cr, As, and Se. After optimizing the temperature-switchable monolithic packing and the automated NIR-actuated speciation scheme, the reliability of this speciation method was validated via analyses of these species in reference materials (CASS-6, SLRS-5, 1643f, and Seronorm Trace Elements Urine L-2) and seawater, river water, groundwater, agricultural wastewater, and human urine samples. The findings indicated that the SPE column with the 4D-printed NIR-actuated temperature-switchable monolithic packing enabled a highly sensitive and reliable speciation analysis of inorganic Cr, As, and Se and provided several advantages, including reduction in operational complexity and improvements in analytical performance and applicability.

## Experimental Section

### Chemicals

NIPAM (415324), *N*,*N*′-methylenebis­(acrylamide) (MBA; 146072), diphenyl­(2,4,6-trimethylbenzoyl)­phosphine oxide (TPO; 415952), disodium hydrogen phosphate (255793, trace metals basis), standard solutions (1000 mg L^–1^) of Cr­(III) (92,966), Cr­(VI) (19036), As­(V) (72718), and Se­(IV) (50,002), arsenic­(III) oxide (202,673), and sodium selenate (71,948) were purchased from Sigma-Aldrich. Graphene nanoplatelets (453,160,500, 2–10 nm) were obtained from Thermo Fisher Scientific. Nitric acid (HNO_3_; 6901, ultrapure grade) was acquired from J.T. Baker. Sodium hydroxide (NaOH; 106,466, Suprapur grade) was purchased from Merck. All solutions were prepared using water purified by a water purification system (Milli-Q IQ 7000; Merck). Working solutions were prepared through serial dilution of stock solutions with 10 mM phosphate buffer, adjusted with 10% HNO_3_ (v/v) and 10% NaOH (w/v) solutions. The NIR-responsive part was fabricated using photocurable resins of thermoresponsive hydrogels, which were prepared by mixing NIPAM, MBA (cross-linker), TPO (photoinitiator), and graphene in ethanol (Figure S1), whereas the nonresponsive part was fabricated using photocurable resins without graphene. Column holders were fabricated using Aqua Clear resins (Phrozen; comprising bisphenol A ethoxylate dimethacrylate, 4-acryloylmorpholine, and TPO).

### SPE Column with the 4D-Printed Temperature-Switchable Monolithic Packing

The SPE column (modeled by SolidWorks 2020, Dassault Systèmes; Figures 1A and S2) was fabricated using a DLP 3D printer (Sonic Mini 8K, Phrozen; *z*-axis resolution: 100 μm) and contained a set of column holders featuring a screw thread pair and a hollow cylindrical chamber for the filling of the monolithic packing. The upper and lower column holders, including the fitting for the standard 10–32 flat-bottom male connector as the loading and outlet ports, were fabricated using Aqua Clear resins. The monolithic packing stacked layer by layer by four cuboids (length: 8.2 mm, width: 0.7 mm, height: 0.7 mm, interstitial space: 1.0 mm) per layer with a twisting angle of 90° (Figure S2) was fabricated with the print–pause–print technique. Initially, the nonresponsive part (14 layers) was fabricated using NIPAM/MBA photocurable resins; subsequently, the resins in the vat were replaced with NIPAM/MBA photocurable resins incorporating graphene (0.4%, w/v) to fabricate the NIR-responsive part (15 layers), allowing *in situ* attachment of the NIR-responsive part to the nonresponsive part without twisting (Figure 1D). Following fabrication [fabrication time: 140 min for holders (curing time: 12 s) and 39 min for the monolithic packing (curing time: 18 s); weight: 6.57 g for holders and 0.30 g for the monolithic packing; total material cost: USD 1.16], the column holders and monolithic packing were assembled (Figures 1B and S3) and washed with 0.5% HNO_3_ solution for 8.0 h to remove uncured resins and contaminants through the two fitted flat-bottom male connectors (P-840 and P-844, IDEX Health & Science) with a short piece of poly­(tetrafluoroethylene) (PTFE) tubing (inner diameter: 0.02 in.).

**Figure 1:**
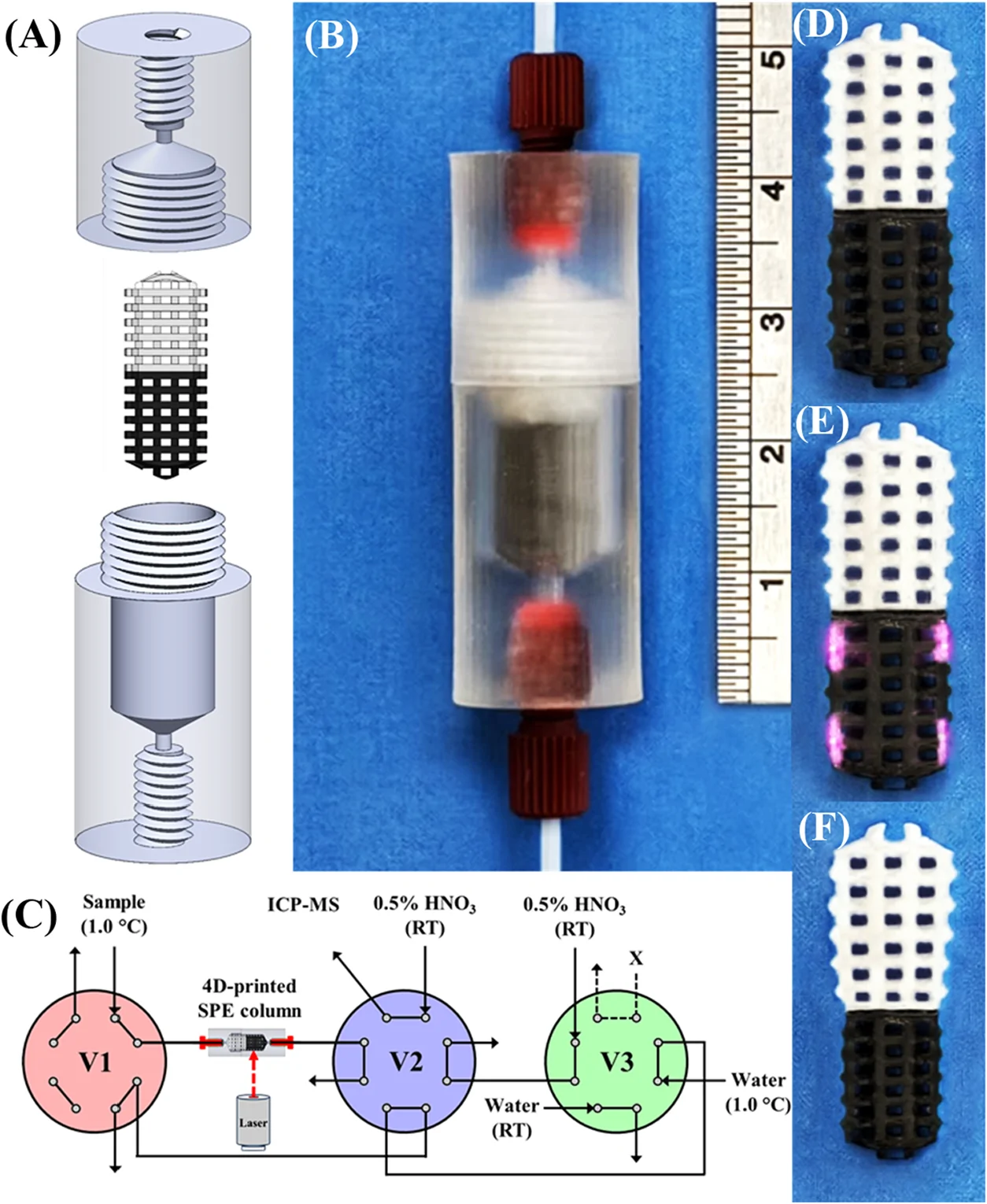
(A) Computer-aided design of the SPE column (gray parts: fabricated by Aqua Clear resins; white part: fabricated by NIPAM/MBA resins; black part: fabricated by graphene-incorporated NIPAM/MBA resins). (B) Photograph of the SPE column fitted with two flat-bottom male connectors. (C) Schematic representation of the automated speciation system. (D) Photograph of the monolithic packing (D) featuring the half nonresponsive part (white) attaching to the graphene-incorporated part (black), (E) with NIR irradiation of the graphene-incorporated part, and (F) with the deswelling of the graphene-incorporated part. V1, V2, and V3: two-position, eight-port rotary valves; arrows: outflow of liquid waste.

### Methods and Apparatus

The NIR-actuated speciation scheme involved six steps (Figures S4 and S5, and Table S1). First, the conditioned sample [1.0 °C, pH 2.0 (10 mM phosphate buffer)] was loaded into the column (flow rate: 1.0 mL min^–1^, sample volume: 1.5 mL) for the selective extraction of Cr­(III), As­(III), and Se­(IV) by the nonresponsive part and Cr­(VI), As­(V), and Se­(VI) by the graphene-incorporated part (with NIR irradiation; Figures 1F and S5A; step 1). The column was rinsed with a water stream (1.0 °C, flow rate: 1.0 mL min^–1^, rinsing volume: 2.0 mL) to remove nonretained species and residual sample matrices from the column (without NIR irradiation; Figure S5B; step 2). The sample and rinsing solutions were maintained at 1.0 °C with a stopped-flow step (320 s) to accelerate the swelling of the graphene-incorporated part and the desorption of Cr­(VI), As­(V), and Se­(VI) (without NIR irradiation; Figure S5C; step 3). For time-resolved analysis (integration time: 50 ms) and external calibration using elution peak areas at *m*/*z* 52 (Cr), 75 (As), and 82 (Se), desorbed species were delivered into an ICP-MS system (Agilent 7700x, Agilent Technologies), which was equipped with a MicroMist nebulizer (AR35–1-FM04EX, Glass Expansion) fitted to a Scott-type quartz double-pass spray chamber and Pt sampling and skimmer cones, using a water stream (at room temperature, flow rate: 1.0 mL min^–1^, elution volume: 1.5 mL; Figure S5D; step 4). The remaining Cr­(III), As­(III), and Se­(IV) on the nonresponsive part were eluted using 0.5% (v/v) HNO_3_ (at room temperature, flow rate: 1.0 mL min^–1^; elution volume: 1.5 mL) and transported to the ICP-MS system for time-resolved analysis (Figure S5E; step 5A), followed by NIR irradiation to induce the deswelling of the graphene-incorporated part for the next extraction (Figures 1E and S5F; step 5B). Finally, the residual eluent in the column was replaced by a water stream (at room temperature, flow rate: 1.0 mL min^–1^; rinsing volume: 0.5 mL) for the next sample loading (Figure S5G; step 6).

The NIR-actuated speciation scheme was fully automated by integrating one 4D-printed SPE column, three eight-port valves [C22Z-3188, Valco; programmed through a serial valve interface (SIV-110, Valco)], a peristaltic pump (MINIPULS 3, Gilson), an ICP-MS system, and a four-channel fiber-coupled laser system (FC-808, CNI Laser; 808 nm) equipped with fibers and collimators (FA10866; spot size: 1.0 mm at 10 cm) and controlled by Laser Device Control software version 1.0 (Figure 1C). NIR irradiation was applied by positioning four spots on both sides of the graphene-incorporated part of the monolithic packing (Figures 1E and S3B) to prevent overheating and ensure a uniform deswelling. Eye protection goggles were worn at all times during the experiments. The analytical performance of a conventional two-column SPE-based elemental speciation scheme was assessed by an automated system developed using two SPE columns with polyNIPAM monolithic packing without incorporating graphene, five eight-port valves, two peristaltic pumps, and an ICP-MS system (Figure S6 and Table S2).

### Sample Collection and Preparation

The analytical reliability of the NIR-actuated speciation method was validated by determining these species in (i) reference materials [CASS-6 (nearshore seawater; National Research Council of Canada), SLRS-5 (untreated river water; National Research Council of Canada),1643f (fresh water; National Institute of Standards and Technology), and Seronorm Trace Elements Urine L-2 (human urine, SERO)], (ii) real seawater, river water, groundwater, agricultural wastewater, and human urine samples [collected via *in situ* filtration using 0.45-μm syringe filters (CHROMAFIL Xtra H-PTFE, Macherey-Nagel)], and (iii) real samples spiked with these species (spiked concentration: 0.25 μg L^–1^). All samples were conditioned to pH 2.0 with 10 mM phosphate buffer (e.g., pH 12.1 for 1643f, pH 1.8 for acidified seawater) at 1.0 °C using a digital chilling–heating dry bath (EchoTherm, Torrey Pines Scientific) and analyzed immediately to minimize species interconversion. Statistical comparisons were performed using Student’s two-tailed unpaired *t*-test, with statistical significance set at *p* < 0.05.

## Results and Discussion

### 4D-Printed Temperature-Switchable Monolithic Packing

High-valent species [namely, Cr­(VI), As­(V), and Se­(VI)] usually exhibited longer retention times than low-valent species [namely, Cr­(III), As­(III), and Se­(IV)] when using reversed-phase liquid chromatography for the speciation analysis of inorganic Cr, As, and Se,^49,50^ suggesting that high-valent species were suitably retained by hydrophobic substances, possibly due to differences in their molecular structures that influence polarity, hydrophobicity, and deprotonation behavior.^3,4,51,52^ For example, Cr­(III) exists as a hydrated cation, whereas Cr­(VI), As­(III), As­(V), Se­(IV), and Se­(VI) are oxyanions; As­(V) and Se­(VI) exhibit more symmetrical structures compared to As­(III) and Se­(IV). The monolithic packing selectively extracted Cr­(III), As­(III), and Se­(IV) (Figure 2A) when the sample temperature (equal to the monolithic packing temperature) was lower than the VPTT of the polyNIPAM hydrogel (33.8 °C; Figure S7)^53–55^ and selectively extracted Cr­(VI), As­(V), and Se­(VI) when the sample temperature was higher than the VPTT, providing a temperature-controlled SPE-based elemental speciation scheme by adjusting the temperature of the sample loaded into the column (or, more precisely, the monolithic packing temperature). The solvent polarity of the samples was found to critically influence the hydrophobic and hydrophilic interactions between these species and the monolithic packing. This was demonstrated by the enhanced extraction of Cr­(III), As­(III), and Se­(IV) by the hydrophilic part (<VPTT), and the suppressed extraction of Cr­(VI), As­(V), and Se­(VI) by the hydrophobic part (>VPTT) as the methanol concentration in the samples increased (the solvent polarity of the samples decreased; Figure 2B).^56,57^ Owing to the efficient light absorption and strong light-to-heat conversion abilities of graphene,^58,59^ NIR irradiation effectively induced an increase in the temperature of the graphene-incorporated polyNIPAM hydrogel (Figure 1E,F) to switch its extraction from Cr­(III), As­(III), and Se­(IV) to Cr­(VI), As­(V), and Se­(VI), followed by Cr­(VI), As­(V), and Se­(VI) desorption by adjusting the sample (or monolithic packing) temperature to be lower than the VPTT. Subsequently, the effect of the monolithic packing design on the signal intensities of these species and the effect of NIR actuation on the induced deswelling–swelling kinetics of the monolithic packing were examined to optimize the NIR-actuated speciation scheme.

**Figure 2:**
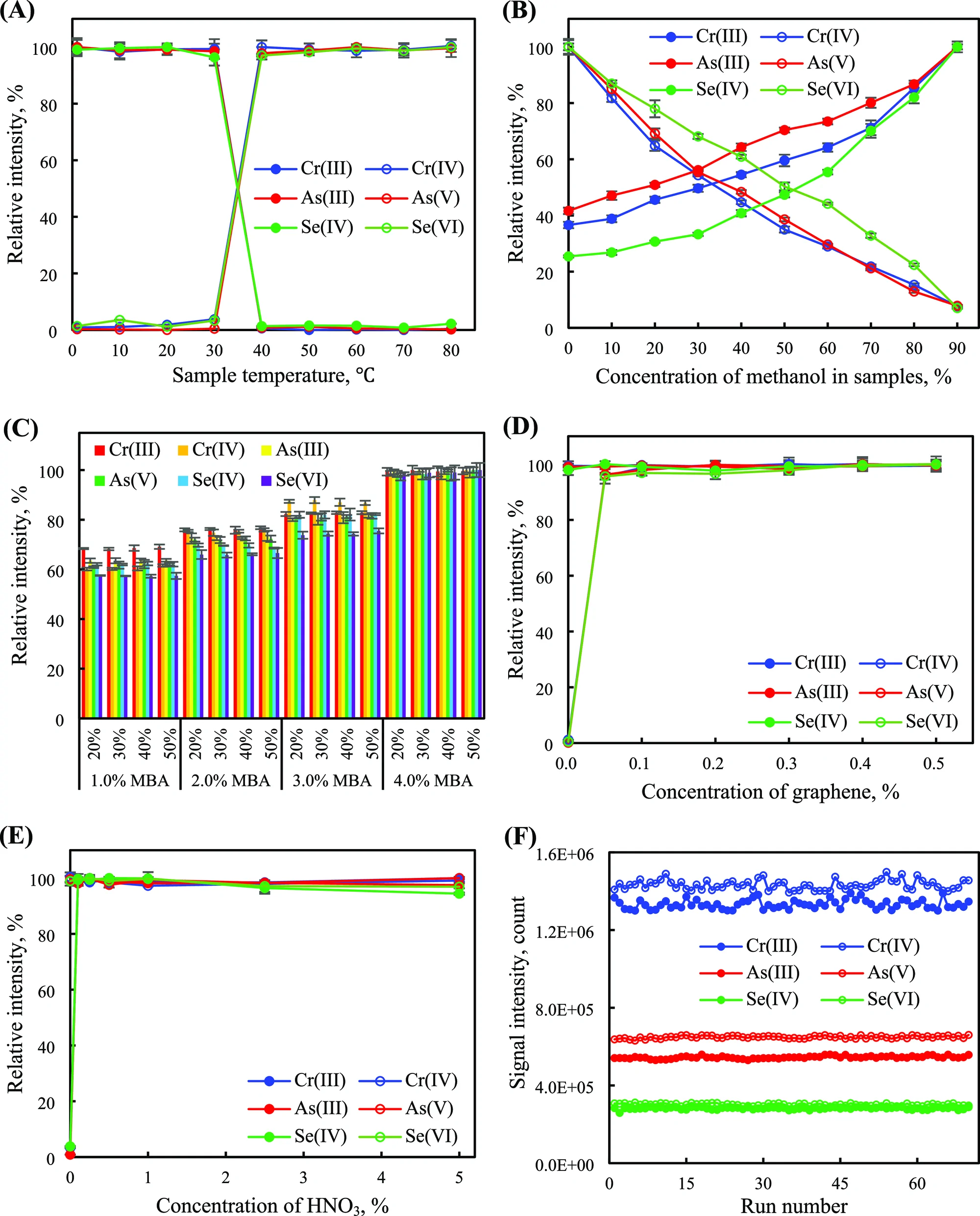
Relative signal intensities of these species plotted with respect to the (A) sample temperature, (B) concentration of methanol in samples, (C) NIPAM and MBA concentrations, (D) concentration of incorporated graphene, and (E) concentration of HNO_3_ in the eluent. (F) Signal intensities of these species over 70 continuous cycles (14.4 h). In (A) and (C)–(F), the concentrations of these species were 1.0 μg L^–1^. In (B), the concentrations of these species were 100 μg L^–1^. Data in (A)–(E) have been normalized to the respective maxima of these species for each parameter. The error bars represent standard deviations (*n* = 6).

The signal intensities of these species increased when the MBA concentration increased because of higher cross-linking density (Figures 2C and S8A); however, the deswelling and swelling times of the monolithic packing became longer when both the NIPAM and MBA concentrations were increased due to the formation of denser hydrogel networks,^60,61^ leading to a lower sample throughput because of the longer NIR-actuated extraction–desorption cycle for Cr­(VI), As­(V), and Se­(VI). Moreover, NIR irradiation enabled an increase in the temperature of the graphene-incorporated part for Cr (VI), As­(V), and Se­(VI) extraction (Figure 2D). Increasing the concentration of the graphene incorporated into the resins reduced the deswelling time of the monolithic packing owing to a stronger photothermal effect (Figure S8B).^61,62^ However, incorporating graphene into the polyNIPAM hydrogel did not alter its ability to extract Cr­(III), As­(III), and Se­(IV) (Figure 2D). Therefore, photocurable resins containing 20% NIPAM and 4.0% MBA without and with graphene (0.4%) were selected to fabricate the nonresponsive part and NIR-actuated thermoresponsive part for the optimal signal intensities of these species and for NIR-induced deswelling–swelling kinetics.

With respect to the monolithic packing design, the signal intensities of these species increased owing to the increase in the surface area of the monolithic packing when the number of cuboids (fixed at a length of 8.2 mm and height of 0.7 mm) per layer was increased from 2 to 4 (at 30 interlacing cuboid layers; Figure S9A), the number of interlacing cuboid layers was increased from 14 to 30 (at 4 cuboids per layer; Figure S9B), and the width of cuboids was increased from 0.5 to 0.7 mm (at 4 cuboids per layer and 30 layers; Figure S9C). However, these decreased when the interstitial spaces between these cuboids were increased from 1.0 to 1.3 mm (Figure S9C), possibly because these species dispersed (were diluted) at a wider interstitial space.^63^ The deswelling and swelling times of the monolithic packing increased when the number of cuboids per layer (Figure S8C), number of interlacing cuboid layers (Figure S8D), and width of cuboids (Figure S8E) were increased possibly due to delays in the transfer of heat and solutions through larger (increased number of cuboids per layer), longer (increased number of interlacing cuboid layers), and denser (increased width of cuboids with the same interstitial space) monolithic packings.^64,65^ Conversely, the deswelling and swelling times decreased when the interstitial spaces between cuboids were increased, indicating higher transfer rates of heat and solutions through less dense interlacing cuboids.^64,65^ The monolithic packing stacked by four cuboids (length: 8.2 mm, width: 0.7 mm, height: 0.7 mm, interstitial space: 1.0 mm) per layer with 29 interlacing cuboid layers (14 layers for the nonresponsive part and 15 layers for the NIR-actuated part; one layer from the nonresponsive part was removed to ensure a proper connection between the two parts without twisting to enhance the strength of the monolithic packing) was selected for the optimal signal intensities of these species and for the shorter deswelling and swelling times of the monolithic packing.

The temperature-dependent nanoporous structure of the polyNIPAM hydrogel [average pore diameter/specific surface area: 4.4 nm/142 m^2^ g^–1^ (1.0 °C; without graphene incorporation) and 3.4 nm/40 m^2^ g^–1^ (40 °C; with graphene incorporation)] ^54,55^ is presented in Figure S10 (SEM images) and S11 (N_2_ adsorption–desorption isotherms). The extraction efficiency-corrected adsorption capacities based on the sample (50 mg L^–1^) that provided the saturated signal intensity of each species were 122.2 μg Cr­(III) cm^–2^, 86.3 μg Cr­(VI) cm^–2^, 121.0 μg As­(III) cm^–2^, 115.9 μg As­(V) cm^–2^, 75.3 μg Se­(IV) cm^–2^, and 134.0 μg Se­(VI) cm^–2^; these corresponded to the amount of these species extracted from a 1.5 mL sample at concentrations of 749 mg Cr­(III) L^–1^, 529 mg Cr­(VI) L^–1^, 742 mg As­(III) L^–1^, 711 mg As­(V) L^–1^, 462 mg Se­(IV) L^–1^, and 822 mg Se­(VI) L^–1^ (total surface area of the monolithic packing: 18.4 cm^2^), respectively.

#### Automated NIR-Actuated Speciation Scheme Using One SPE Column

After optimizing the monolithic packing design, the hydronium ions slightly altered the interactions between the polyNIPAM hydrogel (including positively charged N atoms and negatively charged C=O groups) and elemental species (Cr^3+^, H_2_CrO_4_^–^, H_3_AsO_3_, H_3_AsO_4_, H_2_AsO_4_^–^, H_2_SeO_3_, HSeO_3_^–^, HSeO_4_^–^, SeO_4_^2–^; modeled using Visual MINTEQ version 4.0 ^66^), leading to the pH-dependent signal intensities of these elemental species [nonresponsive part for Cr­(III), As­(III), and Se­(IV) extraction and graphene-incorporated part for Cr­(VI), As­(V), and Se­(VI) extraction; Figure 3A–C].^29,33,67^ Under an acidic sample condition (pH 2.0, 1.0 °C) with a rinsing volume of 2.0 mL (Figure S9D–F), the nonresponsive part (without NIR irradiation; blue lines) selectively extracted Cr­(III), As­(III) (H_3_AsO_3_), and Se­(IV) (H_2_SeO_3_/HSeO_3_^–^) without any contributions from Cr­(VI), As­(V), and Se­(VI), whereas the graphene-incorporated part (with NIR irradiation; red lines) selectively extracted Cr­(VI) (HCrO_4_^–^), As­(V) (H_3_AsO_4_/H_2_AsO_4_^–^), and Se­(VI) (HSeO_4_^–^/SeO_4_^2–^) without any contributions from Cr­(III), As­(III), and Se­(IV) (Figure 3D–F). Thus, the speciation analysis of inorganic Cr, As, and Se was readily performed by determining Cr­(III), As­(III), and Se­(IV) on the nonresponsive part (without NIR irradiation) and Cr­(VI), As­(V), and Se­(VI) on the graphene-incorporated part (with NIR irradiation) at sample pH 2.0 (green lines in Figure 3D–F). The NIR-actuated speciation scheme was automated based on the NIR-induced deswelling of the graphene-incorporated part (Figures 1E,F, and S3B,C) along with sample loading (step 1), elution (step 5B), and rinsing (step 6) steps and on the cold stream-enhanced swelling of the graphene-incorporated part (Figures 1D and S3D) along with rinsing (step 2), stopped-flow (step 3), and elution (steps 4 and 5A) steps.

**Figure 3:**
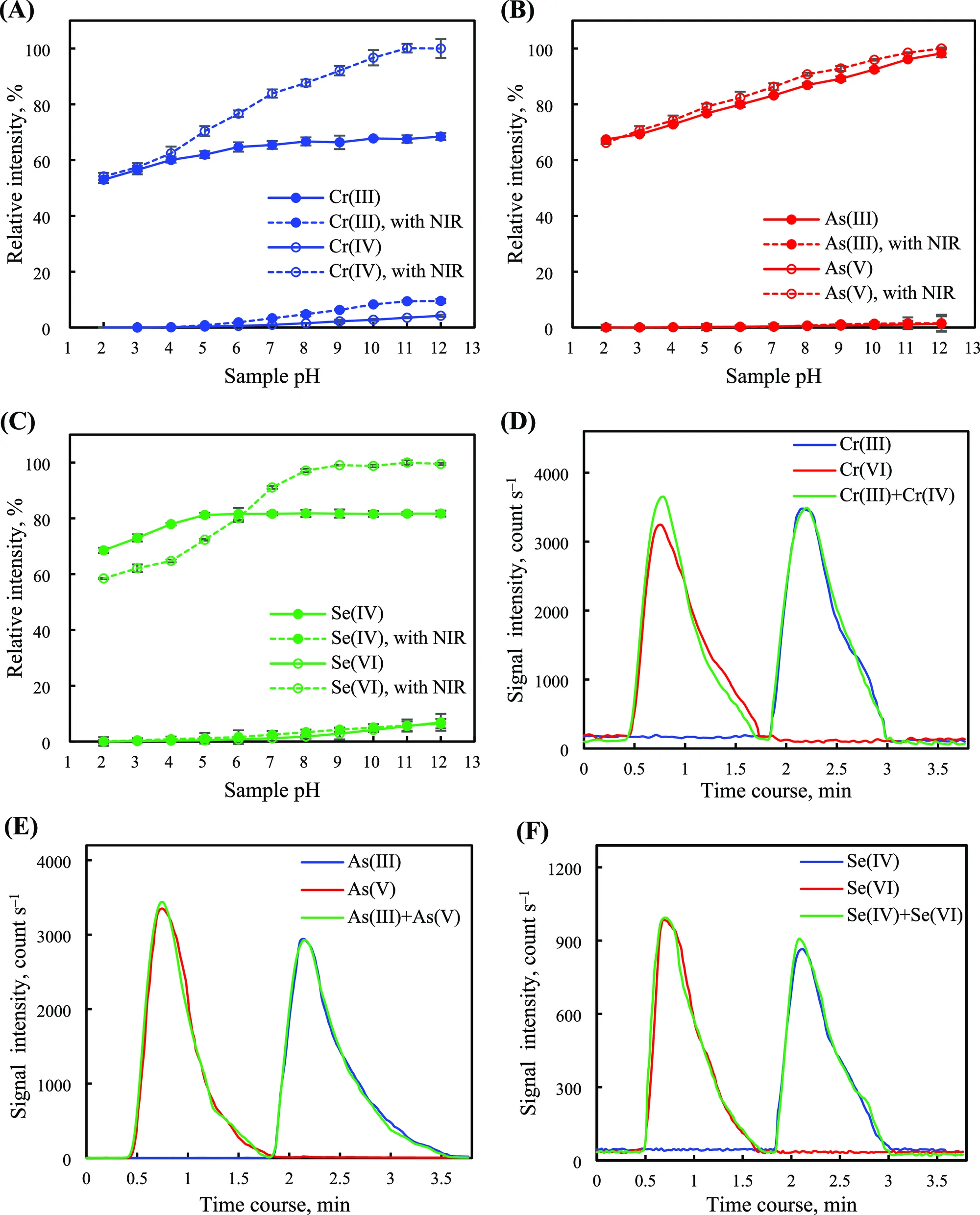
Relative signal intensities of these species plotted with respect to the sample pH for (A) Cr species, (B) As species, and (C) Se species. Elution profiles of (D) Cr species, (E) As species, and (F) Se species. In (A)–(F), the concentrations of these species were 1.0 μg L^–1^. Data in (A)–(C) have been normalized to the respective maxima of these species for each parameter. The error bars represent standard deviations (*n* = 6).

The sample temperature, sample loading flow rate, and sample volume were evaluated to optimize the sample loading step. The sample temperature, which determined not only the interactions between the polyNIPAM hydrogel and elemental species (Figure 2A) but also the swelling time of the graphene-incorporated part after NIR irradiation (Figure S8F), was maintained below the VPTT for Cr­(III), As­(III), and Se­(IV) extraction on the nonresponsive part. The sample and rinsing solutions were fixed at 1.0 °C because they accelerated the swelling of the graphene-incorporated part for Cr­(VI), As­(V), and Se­(VI) desorption. The sample loading flow rate did not influence the signal intensities of these species (>99% of signal intensities maintained at a loading flow rate of 2.0 mL min^–1^ vs those at a loading flow rate of 0.25 mL min^–1^; Figure S9G) owing to the negligible flow resistance from cuboids with 1.0 mm interstitial spaces. However, increasing the sample loading flow rate improved the heat removal rate and decreased the swelling time of the graphene-incorporated part (Figure S8G). A sample loading flow rate of 1.0 mL min^–1^ was selected for a minimal drift in the output flow rate during the aging of peristaltic tubing. Additionally, a sample volume of 1.5 mL was chosen for a higher sample throughput (Figure S9H). The laser power of NIR irradiation was fixed at 6.0 W (laser spot size: 1.0 mm) for shorter deswelling and swelling times (Figure S8H).

The eluent composition, eluent temperature, elution flow rate, and elution volume were assessed for the elution step. Following Cr­(VI), As­(V), and Se­(VI) desorption from the graphene-incorporated part during the stopped-flow step, desorbed species were delivered into an ICP-MS system using a water stream (at room temperature; to facilitate the NIR-induced deswelling of the graphene-incorporated part), which minimized Cr­(III), As­(III), and Se­(IV) desorption from the nonresponsive part (Figure 2E) for elution with a diluted HNO_3_ solution (at room temperature). HNO_3_ concentration (Figure 2E) and eluent temperature (Figure S9I) did not alter the signal intensities of these species. However, the signal intensities increased when the elution volume was increased (Figure S9J). At an elution flow rate of 1.0 mL min^–1^ (Figure S9K), the signal intensities decreased to 5% of the maximum values in 79 s (Figure 3D–F) without obvious carry-over effects (Figure S12A). Accordingly, at a flow rate of 1.0 mL min^–1^, water and 0.5% HNO_3_ were selected as the eluents (1.5 mL) for Cr­(VI), As­(V), and Se­(VI) and for Cr­(III), As­(III), and Se­(IV), respectively. After optimizing the NIR-actuated heating and stream-enhanced cooling of the monolithic packing, its deswelling and swelling times for setting the operating sequence of the NIR-actuated automated speciation system were 82 and 440 s, respectively (Figure S4 and Table S1).

All interdevice variations [relative standard deviations (RSDs) of the signal intensities of these species] from eight columns were <7.3%, confirming the consistency of DLP 4DP in fabricating the monolithic packing. Furthermore, all intradevice variations (RSDs of the signal intensities of these species) from 70 cycles (14.4 h) were <2.0% (Figure 2F), indicating highly reproducible NIR-actuated hydrophilicity–hydrophobicity switching of the monolithic packing and stable extraction–desorption cycle for elemental species. All interday fluctuations (RSDs) of the calibration slopes of these species over 89 days were <15.3% (including calibration curve construction and species analyses in reference materials and real samples; Figure S12B), and no delamination or cracking of the monolithic packing was observed after repeated thermal stress (Figure S13), implying the applicability of the monolithic packing for long-term operation and its resistance to real sample matrices. Common ions [K^+^ (1000 mg L^–1^), Ca^2+^ (1000 mg L^–1^), Mg^2+^ (1000 mg L^–1^), Al^3+^ (100 mg L^–1^), Fe^3+^ (100 mg L^–1^), HCO_3_^–^ (1000 mg L^–1^), SO_4_^2–^ (2000 mg L^–1^), Br^–^ (1000 mg L^–1^), and F^–^ (1000 mg L^–1^); spiked recovery: 98–102%] and dissolved salts (>88% of the signal intensities of these species maintained in a sample containing 4.5% NaCl salinity) did not affect the extraction of these species by the monolithic packing (Figure S9L,M). These results indicated that the SPE column with the 4D-printed NIR-actuated temperature-switchable monolithic packing was consistent, reusable, and durable for the interference-free determination of these species in real samples.

#### Analytical Characteristics

After optimizing the NIR-actuated automated speciation scheme (sample throughput: 4.9 h^–1^; Table S3), all linear correlation coefficients (*R*s) of these species were >0.9998 within a working range of 5.0–500 ng L^–1^ (Figure S12C and Table 1). The extraction efficiencies (ratios of the elution peak areas of these species with and without extraction; 1.0 μg L^–1^) were 87.3–98.8%. The method detection limits (MDLs; three times the standard deviation of baseline noise from seven blank measurements) were 0.1–0.6 ng L^–1^. The enhancement factors (EFs; ratios of the elution peak heights of these species after and before extraction; 1.0 μg L^–1^) were 3.5–6.4. When compared with an automated speciation system (Figure S6 and Table S2) using two SPE columns with the polyNIPAM monolithic packing (without graphene incorporation) and operated at sample temperatures of 1.0 °C [<VPTT for Cr­(III), As­(III), and Se­(IV)] and 40 °C [>VPTT for Cr­(VI), As­(V), and Se­(VI)] (sample throughput: 7.5 h^–1^; Table S4), the *R*s of these species were all >0.9995 (Figure S12D and Table S5), and the MDLs were 0.4–0.8 ng L^–1^. These results indicated that the NIR-actuated speciation system using only one SPE column with the 4D-printed temperature-switchable monolithic packing demonstrated similar analytical performance but required simpler manifolds and fewer valves and pumps for the system’s automation. The quantitative sustainability assessment using the AGREEprep metric indicated that this method was a greener approach with a relatively high level of sustainability (overall greenness score: 0.70; a score above 0.5 indicates a high level of sustainability; Figure S14).^68,69^ Although the sample throughput was lower due to the NIR-actuated deswelling–swelling kinetics of the monolithic packing, our method provided the lowest MDLs and the highest extraction capacities when compared with reported SPE-based elemental speciation schemes for these elemental species (Table S6),^13–74^ and the competitive analytical performance (such as high matrix tolerance and analytical reliability) and cost-effectiveness, characterized by low column cost and high durability, when compared with conventional chromatographic speciation methods. The measured concentrations of these species in the reference materials were consistent with the certified values [relative error: – 1.0% to +2.8%; all *p*-values: >0.7033 (significant difference: *p* < 0.05); all RSDs: <2.8%; spike recoveries: 92–108%], and the spike recoveries of these species in environmental water and human urine samples were 98–103% (Tables S7 and S8), confirming the analytical reliability of our method for determining these species in real environmental water and human urine samples. We introduced NIR actuation to induce the *in situ* switching of the hydrophobicity of the graphene-functionalized polyNIPAM monolithic packing to develop a facile SPE-based elemental speciation scheme as an alternative to conventional two-column SPE-based elemental speciation schemes. It also demonstrated the practical applicability and versatility of 4D-printed devices to minimize complexity and automate SPE-based elemental speciation systems. Considering the switchable hydrophobicity of the 4D-printed polyNIPAM hydrogel, this speciation scheme is expected to be potentially applicable to more elemental species such as Fe­(II) and Fe­(III), Sb­(III) and Sb­(VI), inorganic mercury, and methylmercury.

**Table 1:** Analytical Characteristics of the Automated Speciation System Using the SPE Column with the 4D-Printed NIR-Actuated Temperature-Switchable Monolithic Packing

species	working range, ng L^–1^	calibration curve	*R*	MDL, ng L^–1^	extraction efficiency, %	EF^b^
Cr(III)	5–500	*y* = 313[Cr(III)]^a^ + 413	0.9999	0.1	98.8	5.6
Cr(VI)	5–500	*y* = 336[Cr(VI)]^a^ + 1930	0.9999	0.2	98.7	5.3
As(III)	5–500	*y* = 107[As(III)]^a^ + 323	0.9998	0.3	97.0	6.4
As(V)	5–500	*y* = 82[As(V)]^a^ + 295	1.0000	0.4	98.2	4.0
Se(IV)	5–500	*y* = 40[Se(IV)]^a^ + 182	0.9999	0.6	88.6	3.5
Se(VI)	5–500	*y* = 35[Se(VI)]^a^ + 185	0.9999	0.6	87.3	5.9

a ng L^–1^.

b Ratio of the elution peak heights after and before extraction (1.0 μg L^–1^).

## Conclusions

We developed a highly sensitive, reliable, and easy-to-operate speciation analysis method for inorganic Cr, As, and Se in environmental water and human urine samples by optimizing the NIR-actuated extraction and desorption of these elemental species using one SPE column with 4D-printed NIR-actuated temperature-switchable monolithic packing. As an alternative to conventional two-column SPE-based elemental speciation schemes, it offers the following advantages. First, the NIR-actuated temperature-switchable monolithic packing was 4D-printed without complicated fabrication steps, underscoring the usefulness of 4DP technologies in fabricating versatile stimuli-responsive analytical devices. Second, speciation analysis of inorganic Cr, As, and Se was readily conducted via selective extraction and desorption of these elemental species using the temperature-switchable monolithic packing with and without NIR irradiation. Neither complexing agents nor prereduction or preoxidation of these elemental species is required, suggesting the involvement of fewer parameters when developing the automated speciation system. Third, the property of NIR-actuated *in situ* switching of hydrophobicity enabled the optimization of the speciation analysis of these elements by tuning the deswelling–swelling kinetics of the monolithic packing, highlighting the flexibility of 4DP technologies in improving the performance and applicability of conventional analytical devices. Future studies will focus on optimizing geometric designs (e.g., stacking smaller, ordered cubes or octahedrons) to reduce the deswelling and swelling times of the monolithic packing and to enhance the sample throughput of this speciation method. Most importantly, this study is the first to realize the *in situ* switching of the physicochemical properties of the 4D-printed monolithic packing to fit the separation conditions of these elemental species and to develop a smart nonchromatographic speciation scheme. Although analytical methods based on *in situ* stimuli-activated switching of physicochemical properties of 4D-printed hydrogels remain limited, 4DP technologies will continue to dominate the future development and applications of sample pretreatment schemes in separation science.

## Supplementary Material



## Notes

Detailed dimensions of the SPE column; photographs of the SPE column; photographs of the monolithic packing after repeated deswelling–swelling cycles; time sequence of the NIR-actuated speciation scheme; schematic representation of the automated speciation system; thermal analysis of the graphene-incorporated polyNIPAM hydrogel; deswelling and swelling times of the monolithic packings; relative signal intensities of these species; SEM images of the surfaces of the nonresponsive part and graphene-incorporated part; N_2_ adsorption/desorption isotherms of the nonresponsive part and graphene-incorporated part; carry-over effects of these species in this automated speciation system; daily calibration slopes of these species; calibration plots of these species; operating sequence of the automated speciation system; AGREEprep pictogram displaying the overall greenness score of the automated speciation system; optimized conditions for the automated speciation system; analytical characteristics of the automated speciation system using two SPE columns with the polyNIPAM monolithic packing; analytical characteristics of reported SPE-based elemental speciation methods for inorganic Cr, As, and Se; analytical results of reference materials and environmental water and human urine samples (PDF)

## References

[1] Weekley C. M., Harris H. H. (2013). Which Form is That? The Importance of Selenium Speciation
and Metabolism in the Prevention and Treatment of Disease. Chem. Soc. Rev..

[2] Nearing M. M., Koch I., Reimer K. J. (2014). Complementary Arsenic Speciation
Methods: A Review. Spectrochim. Acta, Part B.

[3] Shahid M., Shamshad S., Rafiq M., Khalid S., Bibi I., Niazi N. K., Dumat C., Rashid M. I. (2017). Chromium Speciation,
Bioavailability, Uptake, Toxicity and Detoxification in Soil-Plant
System: A Review. Chemosphere.

[4] Reid M. S., Hoy K. S., Schofield J. R. M., Uppal J. S., Lin Y., Lu X., Peng H., Le X. C. (2020). Arsenic speciation Analysis: A Review
with an Emphasis on Chromatographic Separations. TrAC,Trends Anal. Chem..

[5] Llaver M., Fiorentini E. F., Oviedo M. N., Quintas P. Y., Wuilloud R. G. (2021). Elemental
Speciation Analysis in Environmental Studies: Latest Trends and Ecological
Impact. Int. J. Environ. Res. Public Health.

[6] de
León C. A. P., Montes-Bayón M., Caruso J. A. (2002). Elemental Speciation
by Chromatographic Separation with Inductively Coupled Plasma Mass
Spectrometry Detection. J. Chromatogr. A.

[7] Marcinkowska M., Barałkiewicz D. (2016). Multielemental
Speciation Analysis by Advanced Hyphenated
Technique – HPLC/ICP-MS: A Review. Talanta.

[8] Yu X., Liu C., Guo Y., Deng T. (2019). Speciation Analysis of Trace Arsenic,
Mercury, Selenium and Antimony in Environmental and Biological Samples
Based on Hyphenated Techniques. Molecules.

[9] Gonzalvez A., Armenta S., Cervera M. L., de la Guardia M. (2010). Non-Chromatographic
Speciation. TrAC,Trends Anal. Chem..

[10] Vieira M. A., Grinberg P., Bobeda C. R. R., Reyes M. N. M., Campos R. C. (2009). Non-Chromatographic
Atomic Spectrometric Methods in Speciation Analysis: A Review. Spectrochim. Acta, Part B.

[11] Gonzalvez A., Cervera M. L., Armenta S., de la Guardia M. (2009). A Review of
Non-Chromatographic Methods for Speciation Analysis. Anal. Chim. Acta.

[12] Welna M., Szymczycha-Madeja A., Pohl P. (2020). Non-Chromatographic
Speciation of
As by HG Technique—Analysis of Samples with Different Matrices. Molecules.

[13] Guerrero M. M. L., Alonso E. V., Pavón J. M. C., Cordero M. T. S., de
Torres A. G. (2012). On-Line Preconcentration using Chelating and Ion-Exchange
Minicolumns for the Speciation of Chromium­(III) and Chromium­(VI) and
Their Quantitative Determination in Natural Waters by Inductively
Coupled Plasma Mass Spectrometry. J. Anal. At.
Spectrom..

[14] Su C. K., Chen W. C. (2018). 3D-printed, TiO_2_ NP–Incorporated
Minicolumn Coupled with ICP-MS for Speciation of Inorganic Arsenic
and Selenium in High-Salt-Content Samples. Microchim.
Acta.

[15] Su C. K., Chen Y. T., Sun Y. C. (2019). Speciation
of Trace Iron in Environmental
Water using 3D-Printed Minicolumns Coupled with Inductively Coupled
Plasma Mass Spectrometry. Microchem. J..

[16] Losev V., Didukh-Shadrina S., Orobyeva A., Borodina E., Elsuf’ev E., Metelitsa S., Ondar U. (2022). Speciation of Inorganic Selenium
in Natural Water by *in situ* Solid-Phase Extraction
using Functionalized Silica. Anal. Methods.

[17] Chen J. R., Tsai W. H., Su C. K. (2023). TiO_2_ Nanoparticle–Coated
3D-Printed Porous Monoliths Enabling Highly Sensitive Speciation of
Inorganic Cr, As, and Se. Anal. Chim. Acta.

[18] Narin I., Kars A., Soylak M. (2008). A Novel Solid
Phase Extraction Procedure
on Amberlite XAD-1180 for Speciation of Cr­(III), Cr­(VI) and Total
Chromium in Environmental and Pharmaceutical Samples. J. Hazard. Mater..

[19] Sigrist M., Albertengo A., Beldoménico H., Tudino M. (2011). Determination of As­(III)
and Total Inorganic As in Water Samples using an On-Line Solid Phase
Extraction and Flow Injection Hydride Generation Atomic Absorption
Spectrometry. J. Hazard. Mater..

[20] Li P., Zhang X.-Q., Chen Y.-J., Lian H.-Z., Hu X. (2014). A Sequential
Solid Phase Microextraction System Coupled with Inductively Coupled
Plasma Mass Spectrometry for Speciation of Inorganic Arsenic. Anal. Methods.

[21] Calderilla C., Maya F., Cerdà V., Leal L. O. (2017). 3D Printed Device
Including Disk-Based Solid-Phase Extraction for the Automated Speciation
of Iron using the Multisyringe Flow Injection Analysis Technique. Talanta.

[22] Portugal L. A., Ferrer L., Serra A. M., da Silva D. G., Ferreira S. L. C., Cerdà V. (2015). A Non-Chromatographic Automated System
for Antimony
Speciation in Natural Water Exploiting Multisyringe Flow Injection
Analysis Coupled with Online Hydride Generation – Atomic Fluorescence
Spectrometry. J. Anal. At. Spectrom..

[23] García-Figueroa A., Musil S., Matoušek T. (2022). Non-chromatographic Speciation Analysis
of Tellurium by HG-ICP–MS/MS at Sub ng L^–1^ Concentration in Natural Waters using Ti^III^ as a Pre-Reducing
Agent. Anal. Chem..

[24] He M., Huang L., Zhao B., Chen B., Hu B. (2017). Advanced Functional
Materials in Solid Phase Extraction for ICP-MS Determination of Trace
Elements and Their Species - A Review. Anal.
Chim. Acta.

[25] Herrero-Latorre C., Barciela-García J., García-Martín S., Peña-Crecente R. M. (2018). Graphene and Carbon Nanotubes as
Solid Phase Extraction Sorbents for the Speciation of Chromium: A
Review. Anal. Chim. Acta.

[26] Lorenzo R. A., Carro A. M., Concheiro A., Alvarez-Lorenzo C. (2015). Stimuli-Responsive
Materials in Analytical Separation. Anal. Bioanal.
Chem..

[27] Musarurwa H., Tavengwa N. T. (2022). Stimuli-Responsive Polymers and Their Applications
in Separation Science. React. Funct. Polym..

[28] Abousalman-Rezvani Z., Roghani-Mamaqani H., Riazid H., Abousalman-Rezvani O. (2022). Water Treatment
using Stimuli-Responsive Polymers. Polym. Chem..

[29] Li N., Hou J., Ou R., Yeo L., Choudhury N. R., Zhang H. (2023). Stimuli-Responsive Ion Adsorbents for Sustainable Separation Applications. ACS Nano.

[30] Tokuyama H., Iwama T. (2007). Temperature-Swing Solid-Phase Extraction of Heavy Metals on a Poly­(*N*-isopropylacrylamide) Hydrogel. Langmuir.

[31] Chen J. J., Ahmad A. L., Lim J. K., Ooi B. S. (2019). Adsorption-Desorption
Characteristic of Thermo-Magneto-Responsive Poly­(*N*-isopropylacrylamide)-co-Acrylic Acid Composite Hydrogel Towards
Chromium­(III) Ions. J. Water Process Eng..

[32] Zhang M., Li Y., Yang Q., Huang L., Chen L., Ni Y., Xiao H. (2018). Temperature
and pH Responsive Cellulose Filament/Poly (NIPAM-co-AAc)
Hybrids as Novel Adsorbent Towards Pb­(II) Removal. Carbohydr. Polym..

[33] Cheng J., Shan G., Pan P. (2017). Triple Stimuli-Responsive *N*-isopropylacrylamide Copolymer Toward Metal Ion Recognition
and Adsorption *via* a Thermally Induced Sol–Gel
Transition. Ind. Eng. Chem. Res..

[34] Zhao Z., Zhang Z., Zhu Z., Zou X., Zhao Y., Shi J., Wang J. (2022). Photothermal Responsive
Hydrogel for Adsorbing Heavy
Metal Ions in Aqueous Solution. Colloids Surf.,
A.

[35] Wang W., Liu Z., Wang R., Cao M., Chen Y., Lu X., Ma H., Yue T., Yan T. (2023). A Novel Strategy for Efficient Removal
of Hazardous Metal Ions based on Thermoresponsive Phase Separation
of the PNIPAM/GO System. Chem. Eng. J..

[36] Gross B., Lockwood S. Y., Spence D. M. (2017). Recent
Advances in Analytical Chemistry
by 3D Printing. Anal. Chem..

[37] Nesterenko P. N. (2020). 3D Printing
in Analytical Chemistry: Current State and Future. Pure Appl. Chem..

[38] Carrasco-Correa E. J., Simó-Alfonso E. F., Herrero-Martínez J. M., Miró M. (2021). The Emerging Role of 3D Printing in the Fabrication
of Detection Systems. TrAC, Trends Anal. Chem..

[39] Su C. K. (2021). Review
of 3D-Printed Functionalized Devices for Chemical and Biochemical
Analysis. Anal. Chim. Acta.

[40] Ryan K. R., Down M. P., Banks C. E. (2021). Future
of Additive Manufacturing:
Overview of 4D and 3D Printed Smart and Advanced Materials and Their
Applications. Chem. Eng. J..

[41] Wan X., Xiao Z., Tian Y., Chen M., Liu F., Wang D., Liu Y., Bartolo P. J. D. S., Yan C., Shi Y., Zhao R. R., Qi H. J., Zhou K. (2024). Recent Advances
in 4D Printing of Advanced Materials and Structures for Functional
Applications. Adv. Mater..

[42] Ding A., Tang F., Alsberg E. (2025). 4D Printing: A Comprehensive Review
of Technologies, Materials, Stimuli, Design, and Emerging Applications. Chem. Rev..

[43] Tariq A., Arif Z. U., Khalid M. Y., Hossain M., Rasool P. I., Umer R., Ramakrishna S. (2023). Recent Advances
in the Additive Manufacturing
of Stimuli-Responsive Soft Polymers. Adv. Eng.
Mater..

[44] Sood N., Bhardwaj A., Mehta S., Mehta A. (2016). Stimuli-Responsive
Hydrogels in Drug Delivery and Tissue Engineering. Drug Delivery.

[45] Kesharwani P., Bisht A., Alexander A., Dave V., Sharma S. (2021). Biomedical
Applications of Hydrogels in Drug Delivery System: An Update. J. Drug Delivery Sci. Technol..

[46] Li Z., Zhou Y., Li T., Zhang J., Tian H. (2022). Stimuli-Responsive
Hydrogels: Fabrication and Biomedical Applications. View.

[47] Islam M. R., Lu Z., Li X., Sarker A. K., Hu L., Choi P., Li X., Hakobyan N., Serpe M. J. (2013). Responsive Polymers for Analytical
Applications: A Review. Anal. Chim. Acta.

[48] Hrušková H., Řemínek R., Foret F. (2025). Recent Progress in
3D and 4D Printing in Analytical Chemistry with a Focus on Sample
Preparation. TrAC,Trends Anal. Chem..

[49] Do B., Robinet S., Pradeau D., Guyon F. (2021). Speciation of Arsenic
and selenium compounds by Ion-Pair Reversed-Phase Chromatography with
Electrothermic Atomic Absorption Spectrometry. J. Chromatogr. A.

[50] Spinazzè A., Spanu D., Bella P. D., Corti C., Borghi F., Fanti G., Cattaneo A., Wise W. R., Davis S. J., Cavallo D. M., Recchia S. (2022). On the Determination
of Cr­(VI) in
Cr­(III)-Rich Particulates: From the Failure of Official Methods to
the Development of an Alternative Protocol. Int. J. Environ. Res. Public Health.

[51] Krivovichev V. G., Krivovichev S. V., Charykova M. V. (2019). Selenium Minerals: Structural and
Chemical Diversity and Complexity. Minerals.

[52] Rasool A., Pertile E., Brožová K., Halfar J., Čabanová K., Malíková P., Chromíková J., Motyka O., Drabinová S., Heviánková S. (2026). Mechanistic Insights and Environmental
Ramifications of Cr­(III) Oxidation to Cr­(VI) in Soil and Groundwater
Systems: Bridging Geochemical Mechanisms and Emerging Remediation
Strategies. Environ. Geochem. Health.

[53] Halperin A., Krçger M., Winnik F. M. (2015). Poly­(*N*-isopropylacrylamide)
Phase Diagrams: Fifty Years of Research. Angew.
Chem., Int. Ed..

[54] Lanzalaco S., Armelin E. (2017). Poly­(*N*-isopropylacrylamide) and Copolymers:
A Review on Recent Progresses in Biomedical Applications. Gels.

[55] Tang L., Wang L., Yang X., Feng Y., Li Y., Feng W. (2021). Poly­(*N*-isopropylacrylamide)-Based Smart Hydrogels:
Design, Properties and Applications. Prog. Mater.
Sci..

[56] Türker A. R. (2012). Separation,
Preconcentration and Speciation of Metal Ions by Solid Phase Extraction. Sep. Purif. Rev..

[57] Dewulf B., Binnemans K. (2025). Effect of Polar Organic Solvents
on the Separation
of Rare Earths and Transition Metal Chloride Complexes: Comparison
of Ion Exchange, Extraction Chromatography and Solvent Extraction. RSC Adv..

[58] Zhang E., Wang T., Hong W., Sun W., Liu X., Tong Z. (2014). Infrared-Driving Actuation Based on Bilayer Graphene Oxide-Poly­(*N*-isopropylacrylamide) Nanocomposite Hydrogels. J. Mater. Chem. A.

[59] Cui X., Ruan Q., Zhuo X., Xia X., Hu J., Fu R., Li Y., Wang J., Xu H. (2023). Photothermal Nanomaterials:
A Powerful Light-to-Heat Converter. Chem. Rev..

[60] Yıldız B., Isik B., Kis M. (2002). Synthesis
and Characterization of
Thermoresponsive Isopropylacrylamide–Acrylamide Hydrogels. Eur. Polym. J..

[61] Shi K., Liu Z., Wei Y.-Y., Wang W., Ju X.-J., Xie R., Chu L.-Y. (2015). Near-Infrared Light Responsive Poly­(N-isopropylacrylamide)/Graphene
Oxide Nanocomposite Hydrogels with Ultrahigh Tensibility. ACS Appl. Mater. Interfaces.

[62] Yan Q., Ding R., Zheng H., Li P., Liu Z., Chen Z., Xiong J., Xue F., Zhao X., Peng Q., He X. (2023). Bio-Inspired Stimuli-Responsive
Ti_3_C_2_T_x_/PNIPAM Anisotropic Hydrogels
for
High-Performance Actuators. Adv. Funct. Mater..

[63] Tsai W. H., Su C. K. (2024). 4D-Printed Elution-Peak-Guided Dual-Responsive
Monolithic Packing
for the Solid-Phase Extraction of Metal Ions. Anal. Chem..

[64] Liu W., Wang Z., Serna J. A., Debastiani R., Gomez J. E. U., Lu L., Yang W., Dong Z., Levkin P. A. (2024). Enhancing Temperature
Responsiveness of PNIPAM Through
3D-Printed Hierarchical Porosity. Adv. Funct.
Mater..

[65] Hu J., Xu W., Liang H., Shi J., Tang W., Guo B., Yang J., Zhu L. (2025). Flow and Heat Transfer Characteristics
of 3D Printed Sheet and Solid Triply Periodic Minimal Surfaces Porous
Structures. Sci. Rep..

[66] Gustafsson, J. P. Visual MINTEQ Ver. 4.0. https://vminteq.com/. (accessed March 18, 2026).

[67] Zheng H., Zhang S., Yang C., Yin H., Liu W., Lu K. (2021). Simultaneous Removal of Ni­(II) and
Cr­(VI) from Aqueous Solution by
Froth Flotation using PNIPAM-CS Intelligent Nano-Hydrogels as Collector. J. Mol. Liq..

[68] Wojnowski W., Tobiszewski M., Pena-Pereira F., Psillakis E. (2022). AGREEprep
– Analytical Greenness Metric for Sample Preparation. TrAC, Trends Anal. Chem..

[69] Pena-Pereira F., Tobiszewski M., Wojnowski W., Psillakis E. (2022). A Tutorial
on AGREEprep an Analytical Greenness Metric for Sample Preparation. Adv. Sample Prep..

[70] Huang C. Z., Hu B., He M., Duan J. K. (2008). Organic
and Inorganic Selenium Speciation
in Environmental and Biological Samples by Nanometer-Sized Materials
Packed Dual-column Separation/Preconcentration On-line Coupled with
ICP-MS. J. Mass Spectrom..

[71] Peng H., Zhang N., He M., Chen B. B., Hu B. (2015). Simultaneous
Speciation Analysis of Inorganic Arsenic, Chromium, and Selenium in
Environmental Waters by 3-(2-Aminoethylamino) Propyltrimethoxysilane
Modified Multi-Wall Carbon Nanotubes Packed Microcolumn Solid Phase
Extraction and ICP-MS. Talanta.

[72] Zhao L. Y., Fei J. J., Lian H. Z., Mao L., Cui X. B. (2019). Simultaneous
Speciation Analysis of Chromium and Antimony by Novel Carboxyl-Functionalized
Hybrid Monolithic Column Solid Phase Microextraction Coupled with
ICP-MS. J. Anal. At. Spectrom..

[73] Chen Z., Chen B., He M., Hu B. (2020). Magnetic Metal-Organic
Framework Composites for Dual-Column Solid-Phase Microextraction Combined
with ICP-MS for Speciation of Trace Levels of Arsenic. Microchim. Acta.

[74] Zhao L. Y., Fei J. J., Lian H. Z., Mao L., Cui X. B. (2020). Development
of a Novel Amine- and Carboxyl-Bifunctionalized Hybrid Monolithic
Column for Non-Invasive Speciation Analysis of Chromium. Talanta.

