# Supplementary material for: 4D-Printed Temperature-Switchable Monolithic Packing for NIR-Actuated Elemental Speciation Analysis
Source: Anal Chem. 2026 Aug 26;98(39):28717–27. doi: 10.1021/acs.analchem.6c01832 (PMC13647975; doi:10.1021/acs.analchem.6c01832)
Supplement: Supplementary file 1 [file ac6c01832_si_001.pdf]

**Supporting Information**

# 4D-Printed Temperature-Switchable Monolithic Packing for NIR-Actuated Elemental Speciation Analysis

Yi-Ting Chen, Yi-Chia Ho, and Cheng-Kuan Su*

Department of Chemistry, National Chung Hsing University, Taichung City 40227, Taiwan (R.O.C.)

**Corresponding Author**

Cheng-Kuan Su

E-mail: cksu@nchu.edu.tw

# Table of Content


| | |
|---|---|
| Page S-5 | **Figure S1.** Infrared spectra of the graphene-incorporated polyNIPAM hydrogel, recorded using a Fourier transform infrared spectrometer. |
| Page S-6 | **Figure S2.** Detailed dimensions of the SPE column. |
| Page S-7 | **Figure S3.** Photographs of the SPE column with the 4D-printed temperature-switchable monolithic packing. |
| Page S-8 | **Figure S4.** Time sequence of the NIR-actuated speciation scheme using the SPE column with the 4D-printed temperature-switchable monolithic packing, with the programming of NIR irradiation of the graphene-incorporated part. |
| Page S-9 | **Figure S5.** Schematic representation of the automated speciation system using the SPE column with the 4D-printed NIR-actuated temperature-switchable monolithic packing. |
| Page S-12 | **Fig. S6.** Schematic representation of the automated speciation system using two SPE columns with the polyNIPAM monolithic packing. |
| Page S-14 | **Figure S7.** Thermal analysis of the polyNIPAM hydrogel with incorporation of graphene (0.4%), recorded using a thermal analyzer. |
| Page S-15 | **Figure S8.** Deswelling and swelling times of the monolithic packings plotted with respect to the NIPAM and MBA concentrations, concentration of incorporated graphene, number of cuboids per layer, number of layers of interlacing cuboids, cuboid width and interstitial spaces between cuboids, sample temperature, sample loading flow rate, and laser power. |

| | |
|---|---|
| Page S-17 | **Figure S9.** Relative signal intensities of these species plotted with respect to the number of cuboids per layer, number of layers of interlacing cuboids, cuboid width and interstitial spaces between cuboids, rinsing volume for Cr species, rinsing volume for As species, rinsing volume for Se species, sample loading flow rate, sample volume, eluent temperature, elution volume, elution flow rate, concentration of NaCl, and interference ions. |
| Page S-20 | **Figure S10.** SEM images of the surfaces of the nonresponsive part (1.0 °C) and graphene-incorporated part (40 °C), recorded using a JEOL JSM-7800F scanning electron microscope. |
| Page S-21 | **Figure S11.** $N_2$ adsorption/desorption isotherms of the nonresponsive part (1.0 °C) and graphene-incorporated part (40 °C). |
| Page S-22 | **Figure S12.** (A) Carry-over effects of these species in this automated speciation system. (B) Daily calibration slopes of these species for the same column used up to 89 days. (C) Calibration plots of these species in the automated speciation system using one SPE column with the 4D-printed NIR-actuated temperature-switchable monolithic packing. (D) Calibration plots of these species in the automated speciation system using two SPE columns with the polyNIPAM monolithic packing. |
| Page S-23 | **Figure S13.** Photographs of the 4D-printed temperature-switchable monolithic packing after repeated NIR-actuated deswelling–swelling cycles. |
| Page S-24 | **Figure S14.** AGREEprep pictogram displaying the overall greenness score of the automated speciation system using the SPE column with the 4D-printed NIR-actuated temperature-switchable monolithic packing. |

| | |
|---|---|
| Page S-25 | **Table S1.** Operating sequence of the automated speciation system using the SPE column with the 4D-printed NIR-actuated temperature-switchable monolithic packing |
| Page S-26 | **Table S2.** Operating sequence of the automated speciation system using two SPE columns with the polyNIPAM monolithic packing |
| Page S-27 | **Table S3.** Optimized conditions for the automated speciation system using the SPE column with the 4D-printed NIR-actuated temperature-switchable monolithic packing |
| Page S-29 | **Table S4.** Operating conditions for the automated speciation system using two SPE columns with the polyNIPAM monolithic packing |
| Page S-31 | **Table S5.** Analytical characteristics of the automated speciation system using two SPE columns with the polyNIPAM monolithic packing |
| Page S-32 | **Table S6.** Analytical characteristics of reported SPE-based elemental speciation methods for inorganic Cr, As, and Se |
| Page S-35 | **Table S7.** Analytical results of reference materials (*n* = 6) |
| Page S-37 | **Table S8.** Analytical results of environmental water and human urine samples (*n* = 6) |

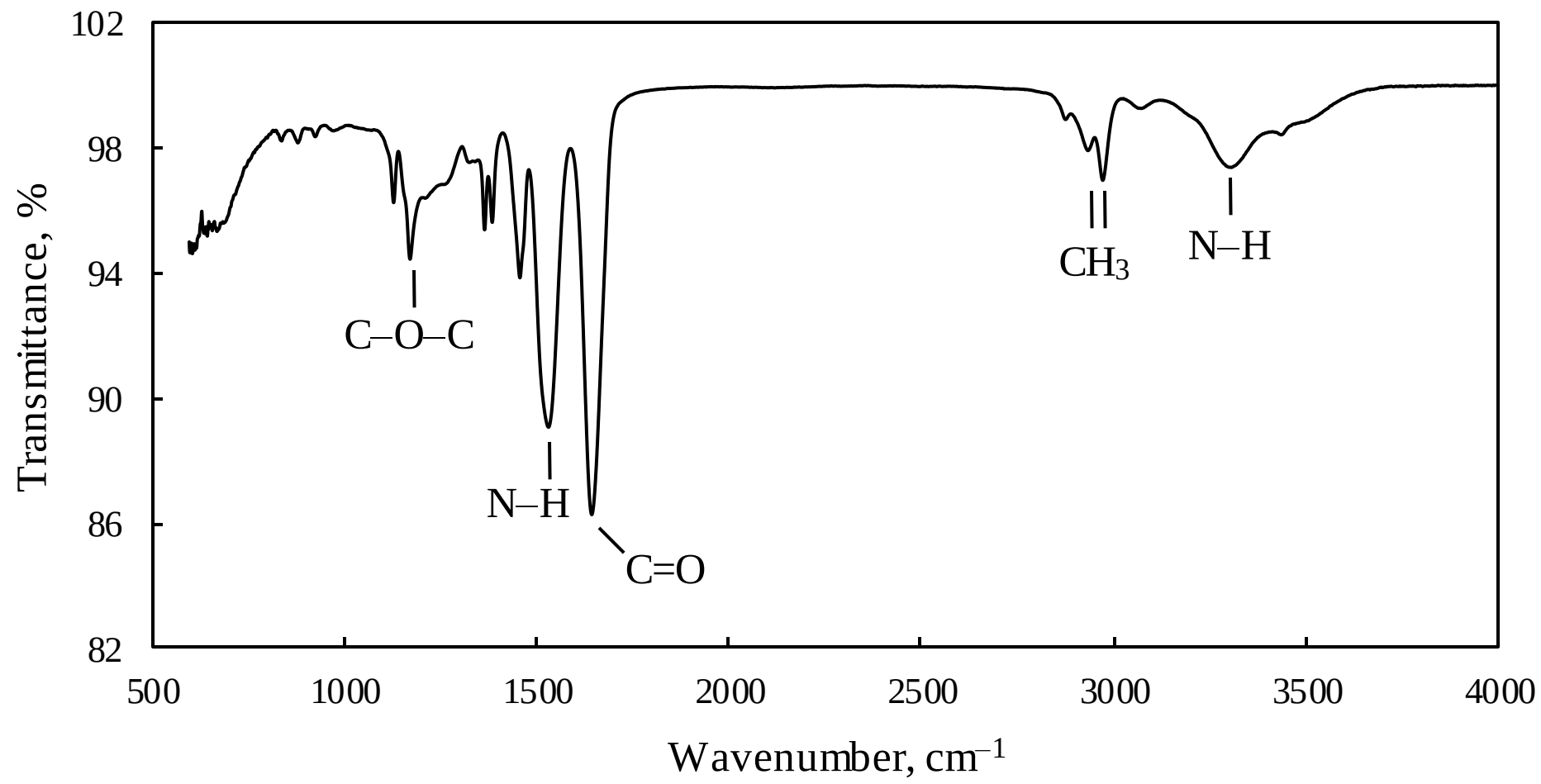


**Figure S1.** Infrared spectra of the graphene-incorporated polyNIPAM hydrogel, recorded using a Fourier transform infrared spectrometer (Tensor 27, Bruker).

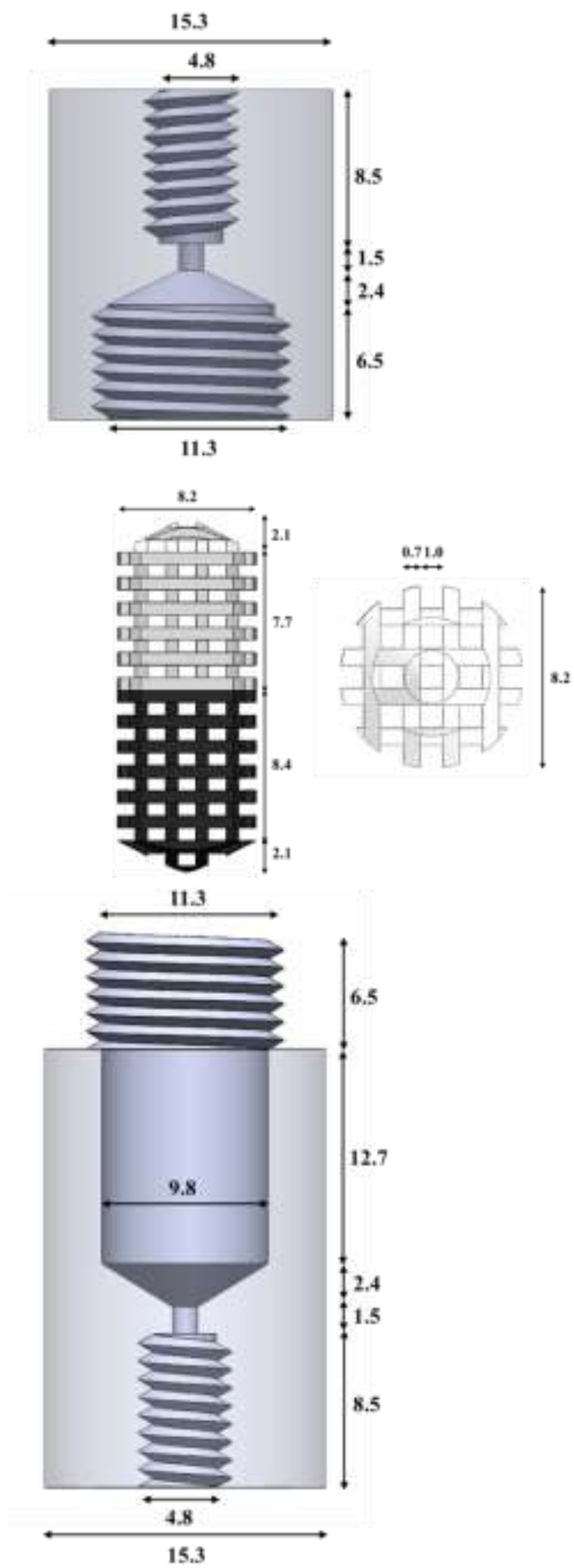


**Figure S2.** Detailed dimensions of the SPE column. The grey parts were fabricated using Aqua Clear resins, the white part was fabricated using NIPAM/MBA resins, and the black was fabricated using graphene-incorporated NIPAM/MBA resins. Units: mm.

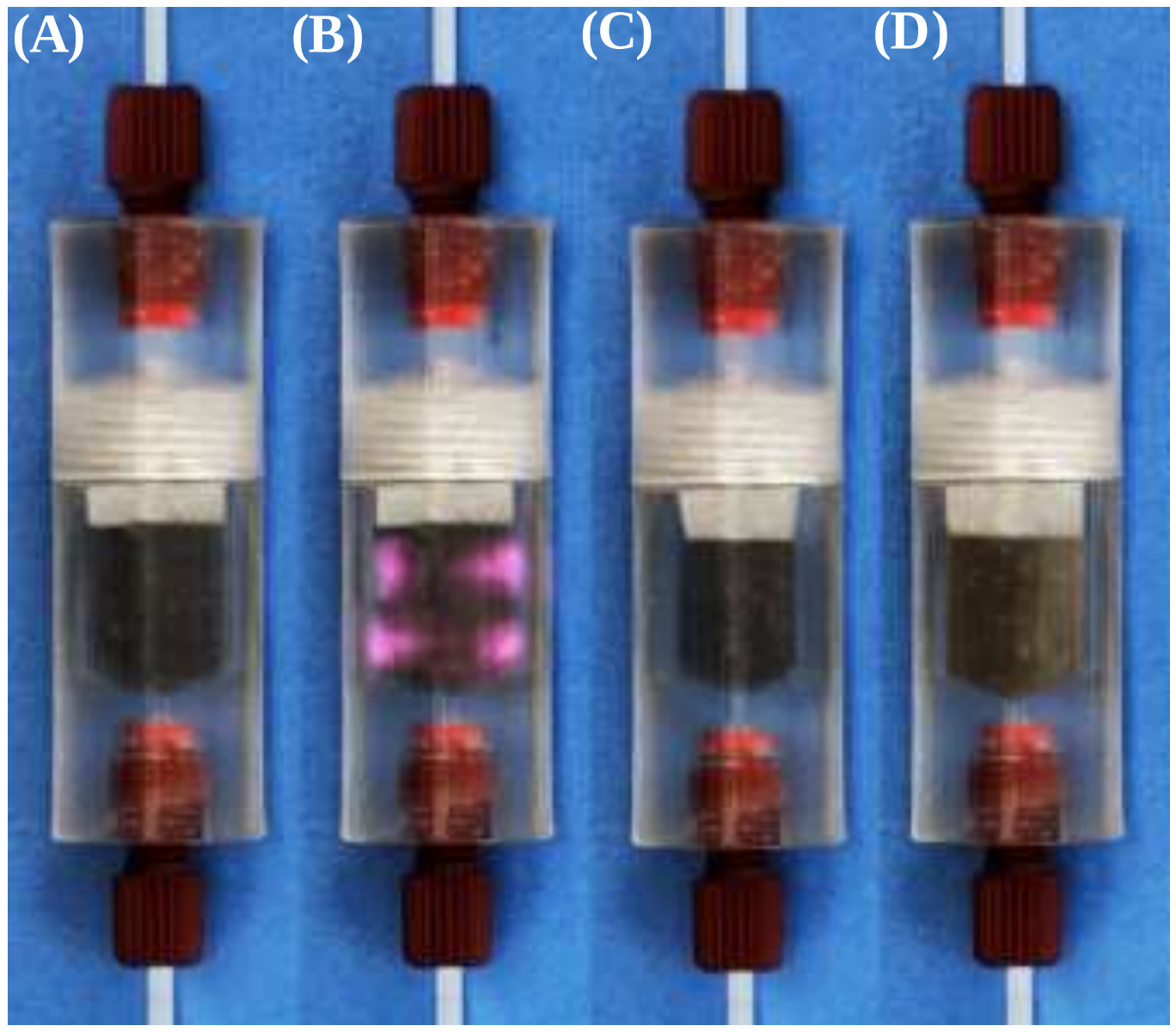


**Figure S3.** Photographs of the SPE column with the 4D-printed temperature-switchable monolithic packing (A) before NIR irradiation of the graphene-incorporated part, (B) during NIR irradiation of the graphene-incorporated part, (C) after NIR irradiation with the deswelling of the graphene-incorporated part, and (D) with the swelling of the graphene-incorporated part.

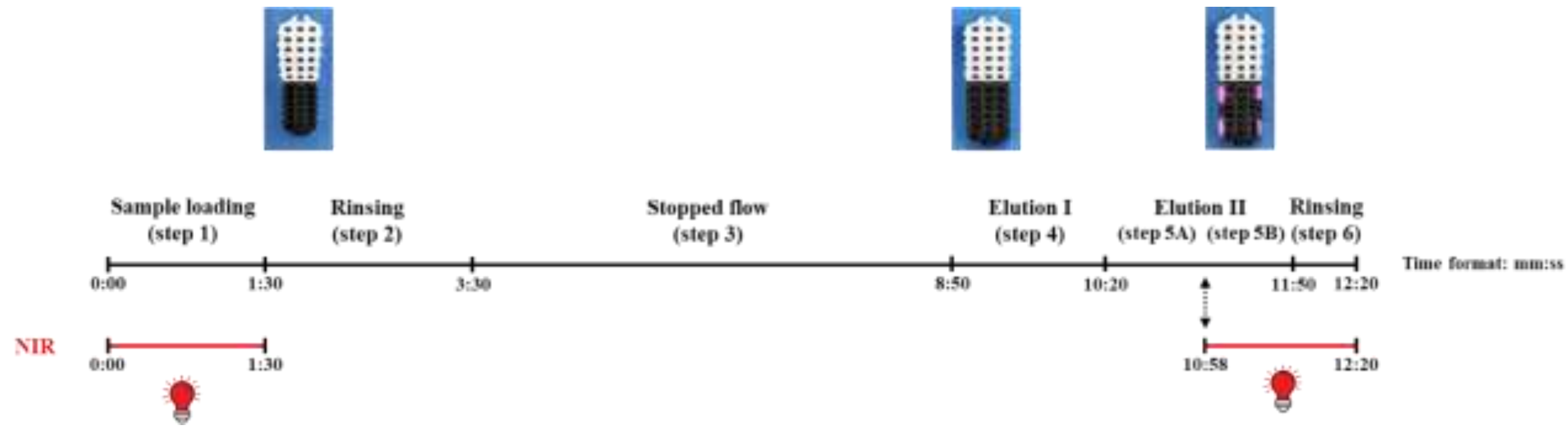


**Figure S4.** Time sequence of the NIR-actuated speciation scheme using the SPE column with the 4D-printed temperature-switchable monolithic packing, with the programming of NIR irradiation of the graphene-incorporated part. Time format: mm:ss.

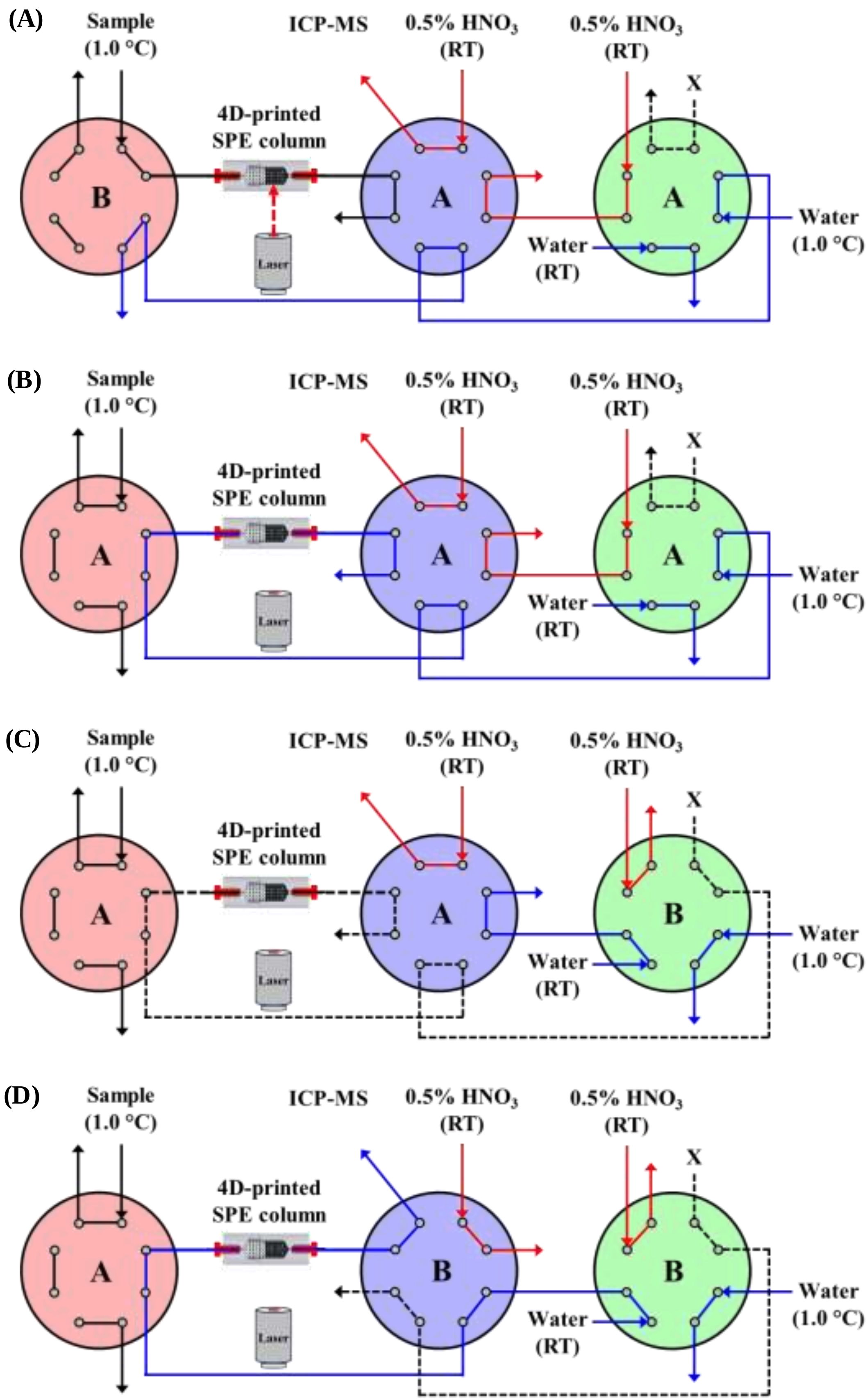
(A)
Sample
(1.0 °C)
ICP-MS
0.5% $HNO_3$
(RT)
0.5% $HNO_3$
(RT)
X
4D-printed
SPE column
B
A
A
Laser
Water
(RT)
Water
(1.0 °C)
(B)
Sample
(1.0 °C)
ICP-MS
0.5% $HNO_3$
(RT)
0.5% $HNO_3$
(RT)
X
4D-printed
SPE column
A
A
A
Laser
Water
(RT)
Water
(1.0 °C)
(C)
Sample
(1.0 °C)
ICP-MS
0.5% $HNO_3$
(RT)
0.5% $HNO_3$
(RT)
X
4D-printed
SPE column
A
A
B
Laser
Water
(RT)
Water
(1.0 °C)
(D)
Sample
(1.0 °C)
ICP-MS
0.5% $HNO_3$
(RT)
0.5% $HNO_3$
(RT)
X
4D-printed
SPE column
A
B
B
Laser
Water
(RT)
Water
(1.0 °C)

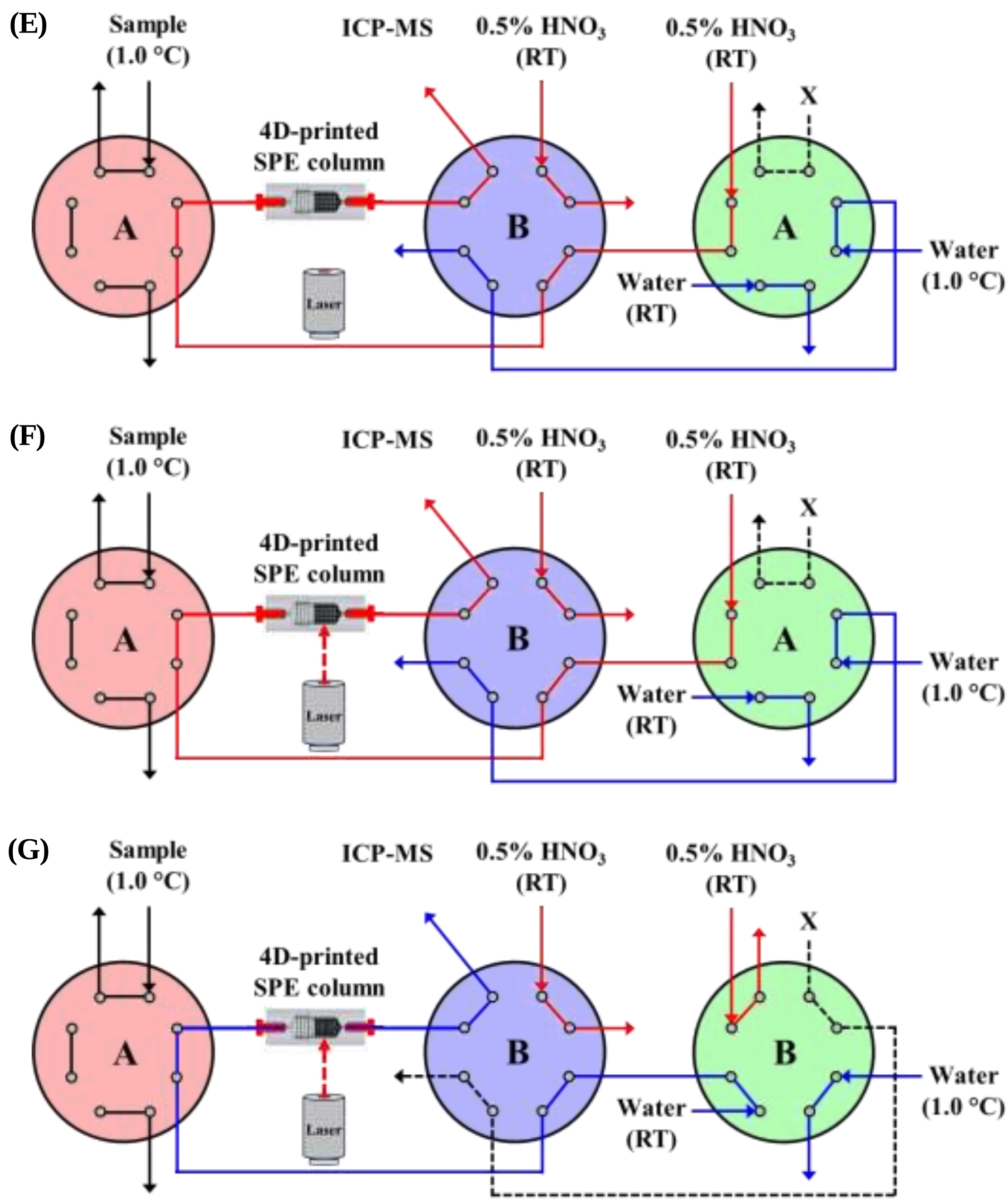


**Figure S5.** Schematic representation of the automated speciation system using the SPE column with the 4D-printed NIR-actuated temperature-switchable monolithic packing. (A) Step 1: The conditioned sample (1.0 °C, pH 2.0) was loaded (1.0 mL min$^{-1}$) into the SPE column for selective extraction of Cr(III), As(III) and Se(IV) by the nonresponsive part and Cr(VI), As(V), and Se(VI) by the graphene-incorporated part (with NIR irradiation). (B) Step 2: A water stream (1.0 °C, flow rate: 1.0 mL min$^{-1}$) was used to rinse the column for removing non-retained species and residual

sample matrices (without NIR irradiation). (C) Step 3: A stopped-flow step was used to allow the swelling of the graphene-incorporated part and desorption of Cr(VI), As(V), and Se(VI) (without NIR irradiation). (D) Step 4: The desorbed Cr(VI), As(V), and Se(VI) were delivered into an ICP-MS system using a water stream (room temperature, flow rate: 1.0 mL $min^{-1}$) for time-resolved analysis (without NIR irradiation). (E) Step 5A: The extracted Cr(III), As(III) and Se(IV) on the nonresponsive part were eluted using 0.5% $HNO_3$ (room temperature, flow rate: 1.0 mL $min^{-1}$) and transported into the ICP-MS system for time-resolved analysis (without NIR irradiation). (F) Step 5B: The extracted Cr(III), As(III) and Se(IV) on the nonresponsive part were eluted using 0.5% $HNO_3$ (room temperature, flow rate: 1.0 mL $min^{-1}$) and transported into the ICP-MS system for time-resolved analysis (with NIR irradiation). (G) Step 6: The residual eluent in the column was replaced by a water stream (room temperature, flow rate: 1.0 mL $min^{-1}$) for loading the next sample (with NIR irradiation). V1, V2, and V3: two-position, eight-port electric switching valves; unmarked arrow: outflow of liquid waste.

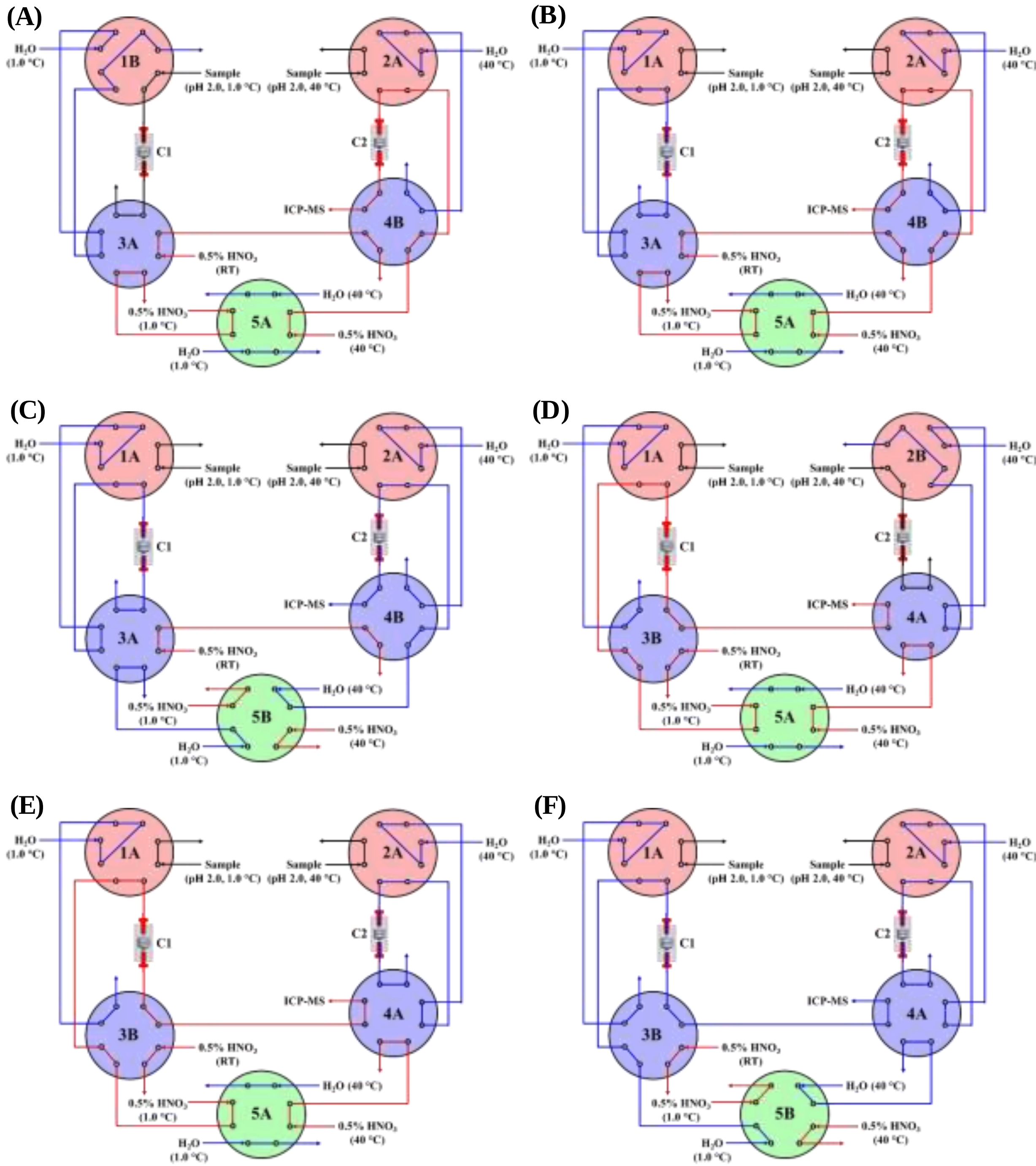


**Fig. S6.** Schematic representation of the automated speciation system using two SPE columns with the polyNIPAM monolithic packing. (A) The conditioned sample (1.0 °C, pH 2.0) was loaded into C1 while the extracted Cr(V), As(V), and Se(VI) in C2 were eluted with 0.5% $HNO_3$ (40 °C) and delivered into the ICP-MS system for time-resolved analysis. (B) The sample loaded in C1 was

rinsed by a pure water stream (1.0 °C) while the extracted Cr(V), As(V), and Se(VI) in C2 were eluted with 0.5% $HNO_3$ (40 °C) and delivered into the ICP-MS system for time-resolved analysis. (C) The sample loaded in C1 was rinsed by a pure water stream (1.0 °C) while the residual eluent in C2 was replaced by another pure water stream (40 °C) for loading of the next sample. (D) The conditioned sample (40 °C, pH 2.0) was loaded into C2 while the extracted Cr(III), As(III), and Se(IV) in C1 were eluted with 0.5% $HNO_3$ (1.0 °C) and delivered into the ICP-MS system for time-resolved analysis. (E) The sample loaded in C2 was rinsed by a pure water stream (40 °C) while the extracted Cr(III), As(III), and Se(IV) in C1 were eluted with 0.5% $HNO_3$ (1.0 °C) and delivered into the ICP-MS system for time-resolved analysis. (F) The sample loaded in C2 was rinsed by a pure water stream (40 °C) while the residual eluent in C1 was replaced by another pure water stream (1.0 °C) for loading of the next sample. C1, C2: SPE columns with the polyNIPAM monolithic packing; V1, V2, V3, V4, and V5: two-position, eight-port rotary valves; unmarked arrow: outflow of liquid waste.

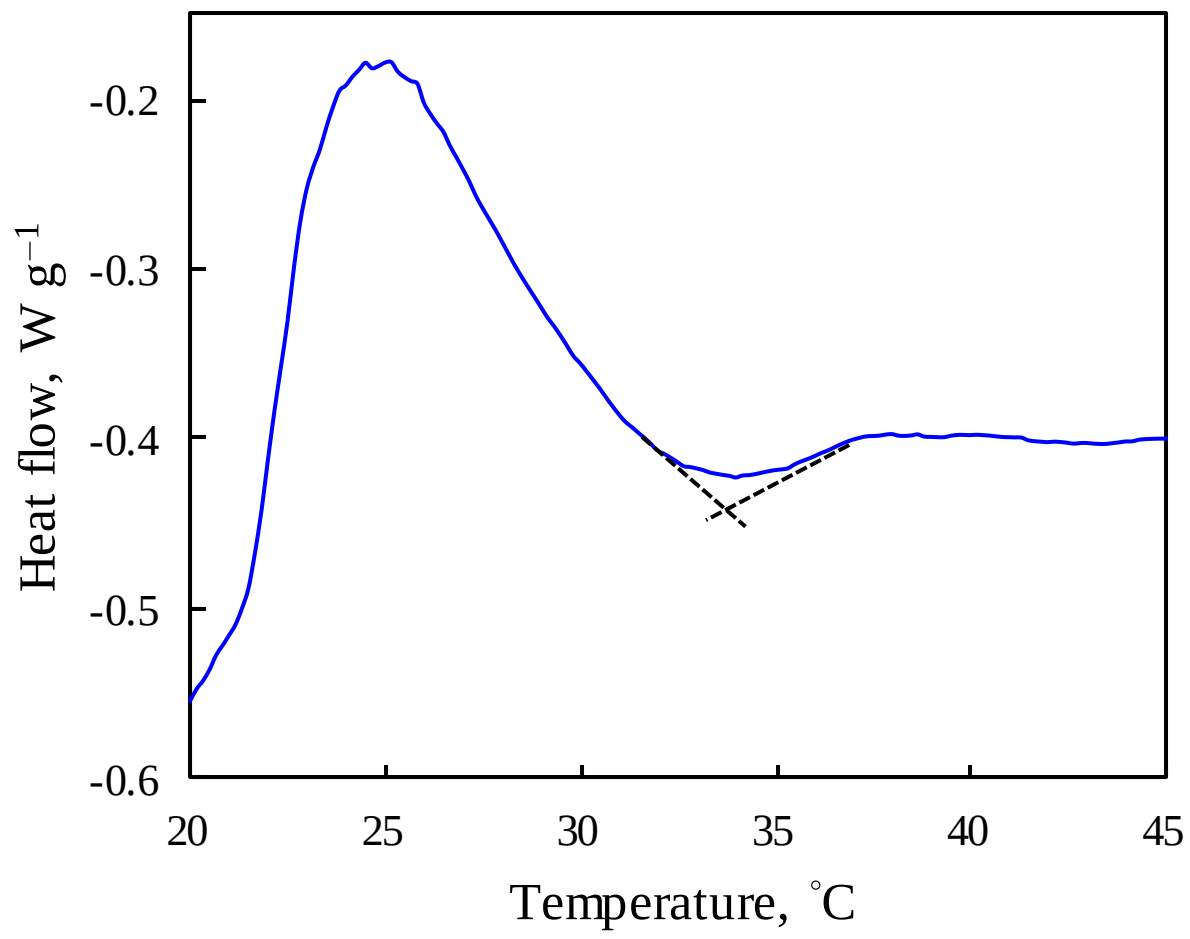


**Figure S7.** Thermal analysis (differential scanning calorimetry) of the polyNIPAM hydrogel with incorporation of graphene (0.4%), recorded using a thermal analyzer (HT-2, Mettler Toledo).

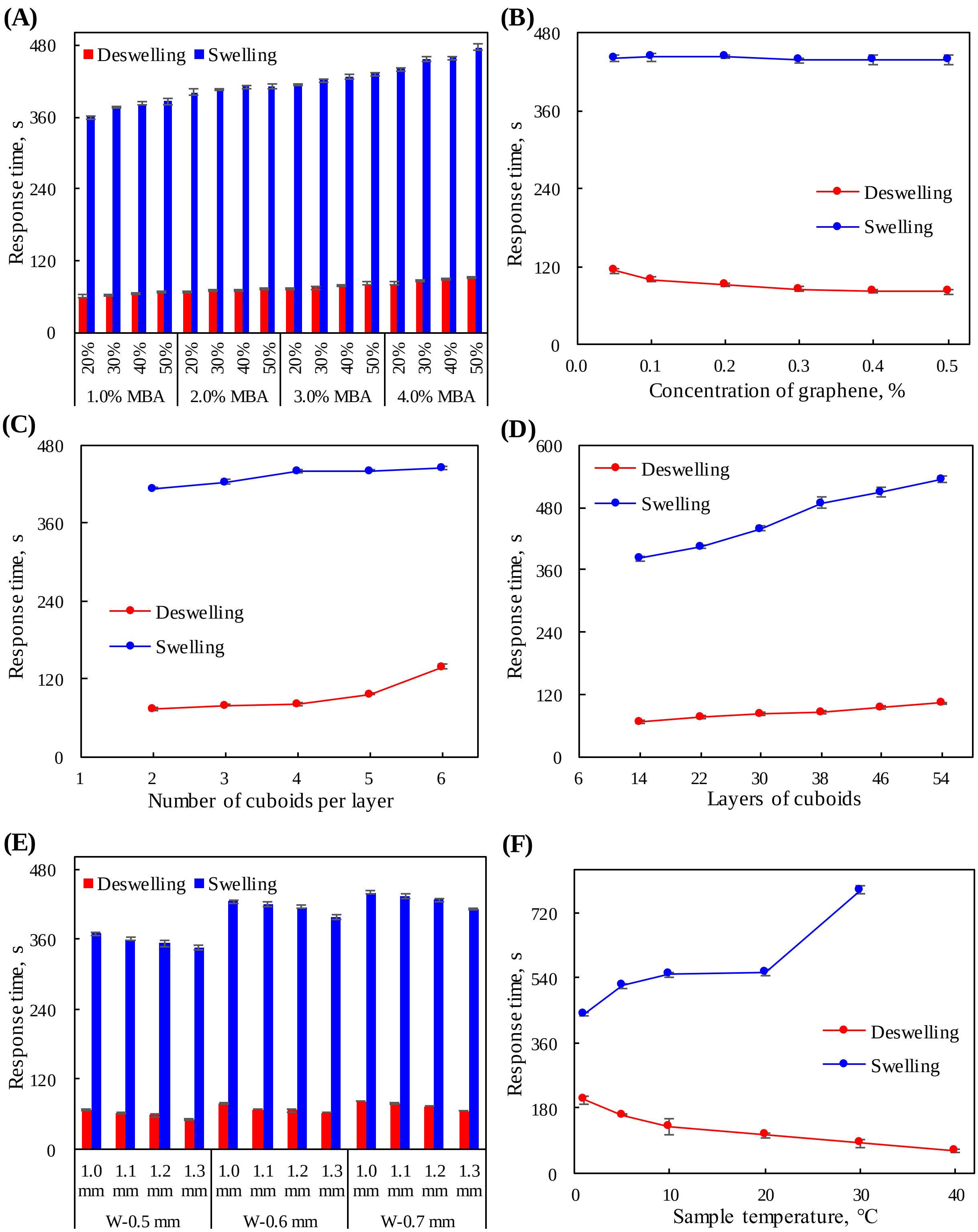

(A)
Deswelling
Swelling
Response time, s
480
360
240
120
0
20%
30%
40%
50%
1.0% MBA
2.0% MBA
3.0% MBA
4.0% MBA
(B)
Deswelling
Swelling
Response time, s
Concentration of graphene, %
0.0
0.1
0.2
0.3
0.4
0.5
(C)
Deswelling
Swelling
Response time, s
Number of cuboids per layer
1
2
3
4
5
6
(D)
Deswelling
Swelling
Response time, s
600
Layers of cuboids
6
14
22
30
38
46
54
(E)
Deswelling
Swelling
Response time, s
1.0 mm
1.1 mm
1.2 mm
1.3 mm
W-0.5 mm
W-0.6 mm
W-0.7 mm
(F)
Deswelling
Swelling
Response time, s
720
540
360
180
0
Sample temperature, °C
0
10
20
30
40

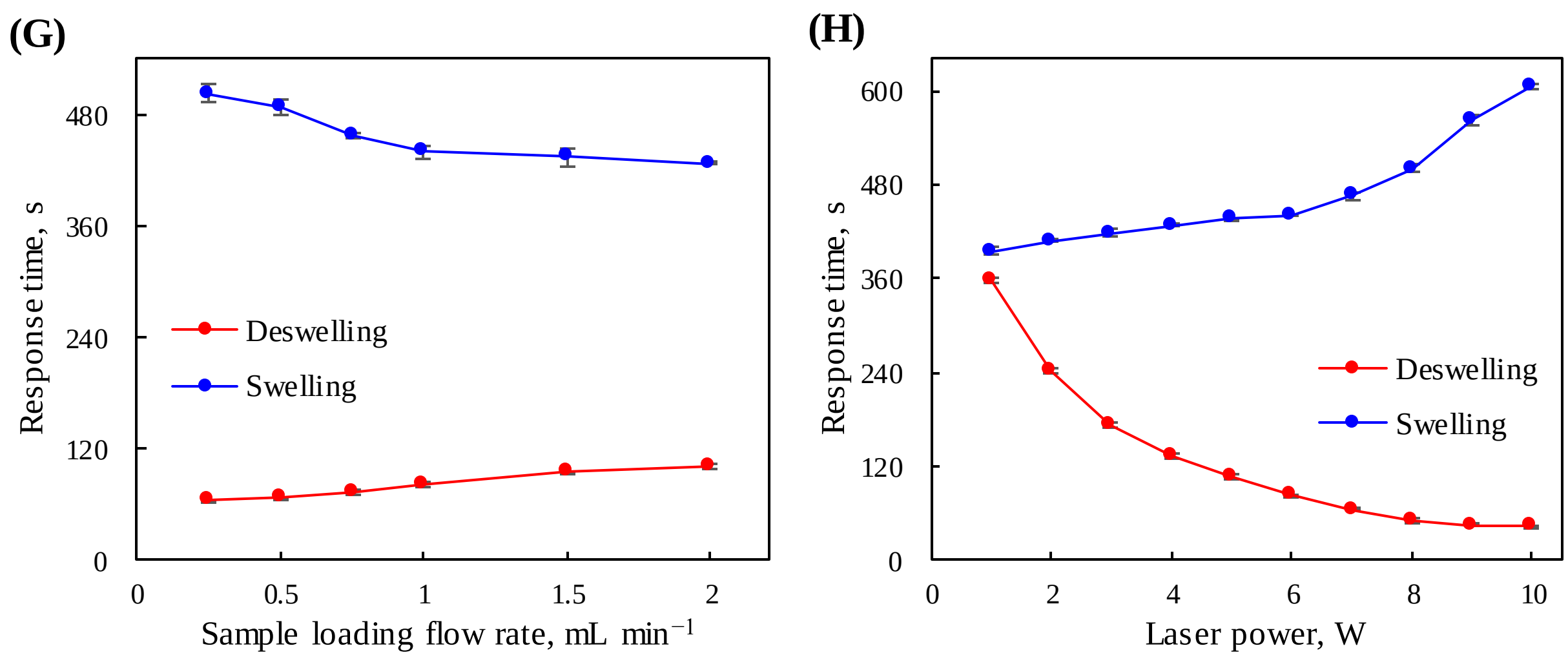


**Figure S8.** Deswelling and swelling times of the monolithic packings plotted with respect to the (A) NIPAM and MBA concentrations, (B) concentration of incorporated graphene, (C) number of cuboids per layer, (D) number of layers of interlacing cuboids, (E) cuboid width and interstitial spaces between cuboids, (F) sample temperature, (G) sample loading flow rate, and (H) laser power. The deswelling times of the monolithic packing were measured from its maximal length to its minimal length when NIR irradiation was initiated; the swelling times of the monolithic packing were measured from its minimal length to its maximal length after stopping NIR irradiation. The error bars represent standard deviations ($n$ = 6).

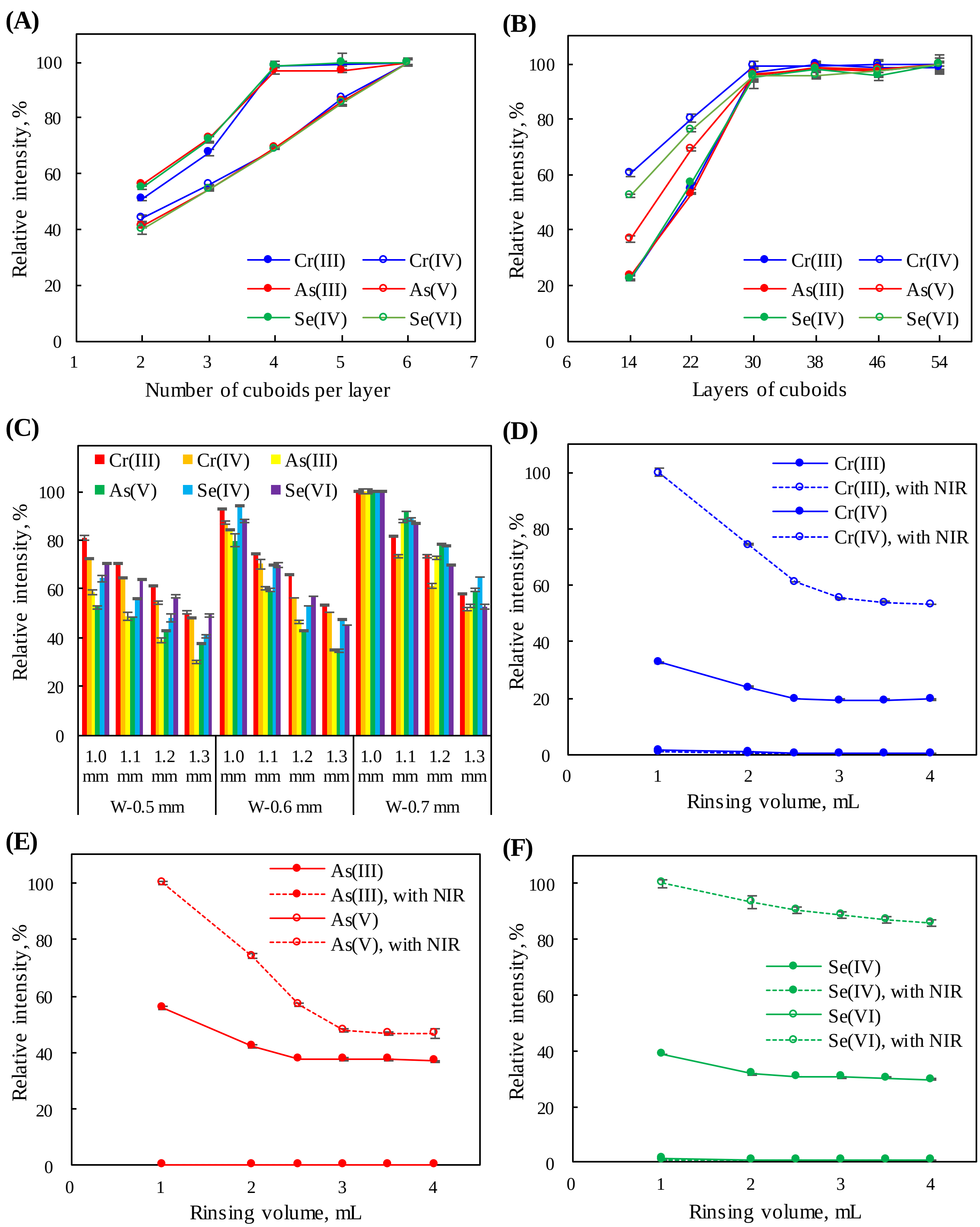

(A)
Relative intensity, %
Number of cuboids per layer
Cr(III)
Cr(IV)
As(III)
As(V)
Se(IV)
Se(VI)
(B)
Relative intensity, %
Layers of cuboids
Cr(III)
Cr(IV)
As(III)
As(V)
Se(IV)
Se(VI)
(C)
Relative intensity, %
Cr(III)
Cr(IV)
As(III)
As(V)
Se(IV)
Se(VI)
1.0 mm
1.1 mm
1.2 mm
1.3 mm
W-0.5 mm
W-0.6 mm
W-0.7 mm
(D)
Relative intensity, %
Rinsing volume, mL
Cr(III)
Cr(III), with NIR
Cr(IV)
Cr(IV), with NIR
(E)
Relative intensity, %
Rinsing volume, mL
As(III)
As(III), with NIR
As(V)
As(V), with NIR
(F)
Relative intensity, %
Rinsing volume, mL
Se(IV)
Se(IV), with NIR
Se(VI)
Se(VI), with NIR

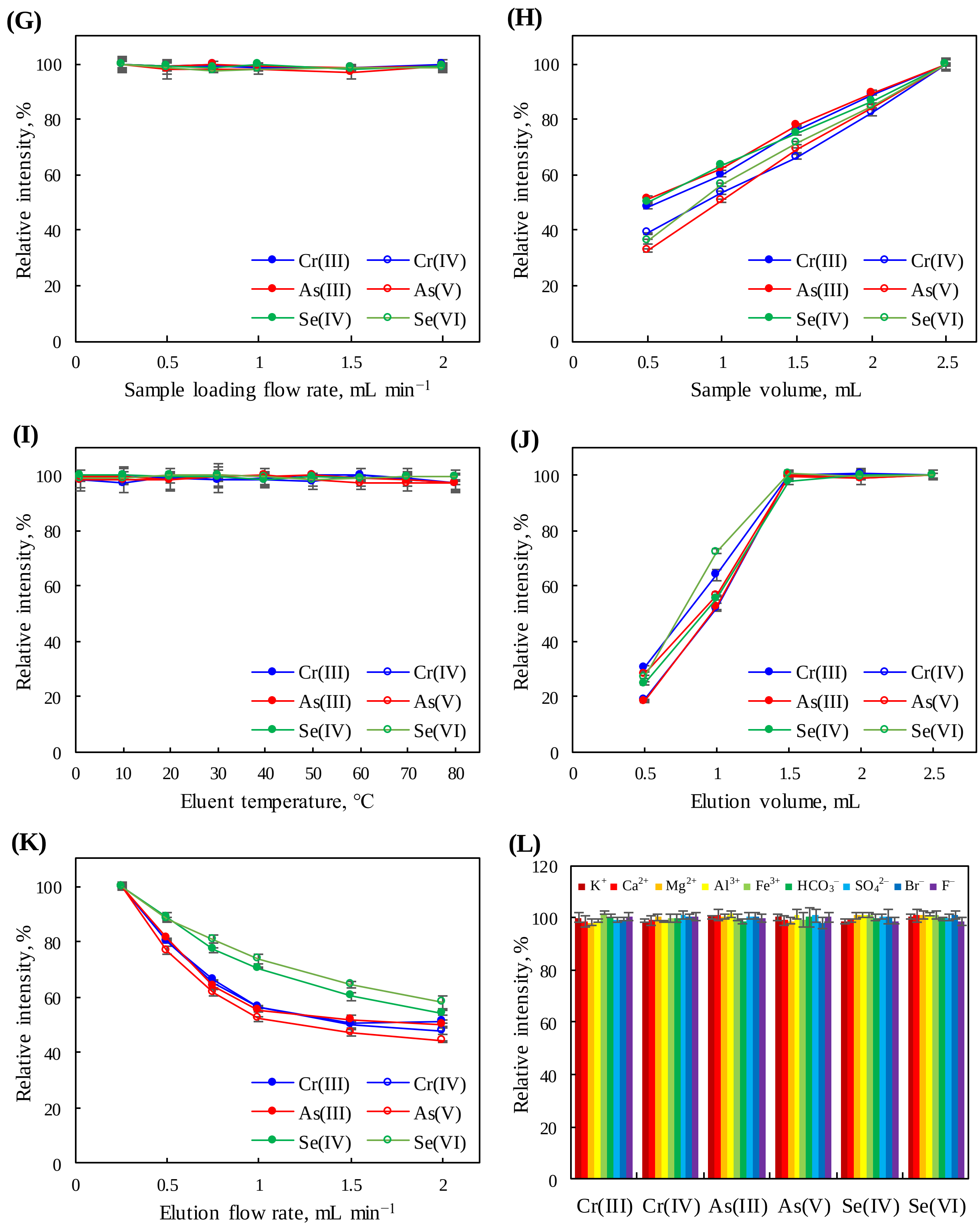
(G)
Relative intensity, %
Sample loading flow rate, mL min$^{-1}$
Cr(III)
Cr(IV)
As(III)
As(V)
Se(IV)
Se(VI)
(H)
Relative intensity, %
Sample volume, mL
(I)
Relative intensity, %
Eluent temperature, °C
(J)
Relative intensity, %
Elution volume, mL
(K)
Relative intensity, %
Elution flow rate, mL min$^{-1}$
(L)
Relative intensity, %
$K^+$ $Ca^{2+}$ $Mg^{2+}$ $Al^{3+}$ $Fe^{3+}$ $HCO_3^-$ $SO_4^{2-}$ $Br^-$ $F^-$
Cr(III) Cr(IV) As(III) As(V) Se(IV) Se(VI)

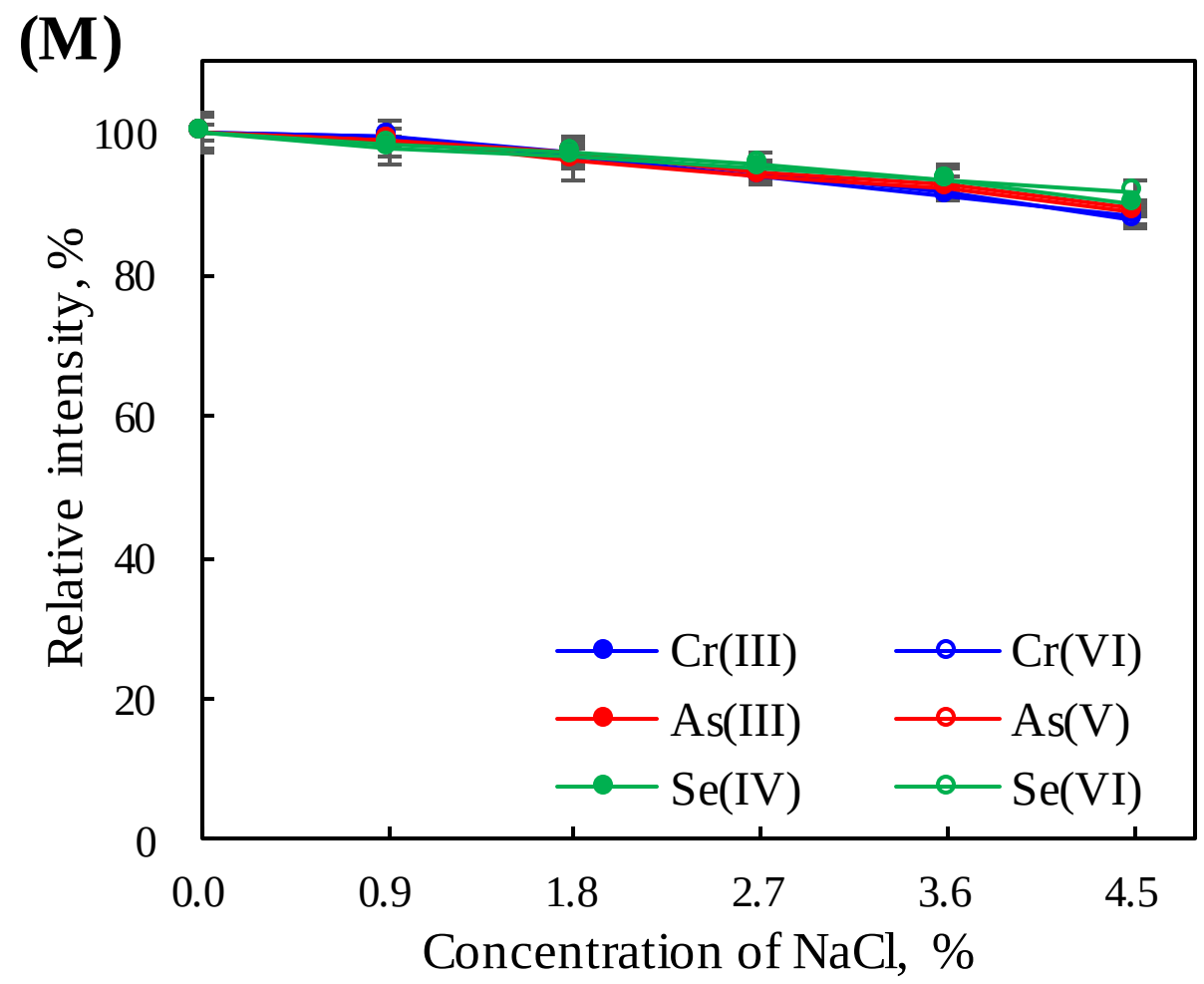


**Figure S9.** Relative signal intensities of these species plotted with respect to the (A) number of cuboids per layer, (B) number of layers of interlacing cuboids, (C) cuboid width and interstitial spaces between cuboids, (D) rinsing volume for Cr species, (E) rinsing volume for As species, (F) rinsing volume for Se species, (G) sample loading flow rate, (H) sample volume, (I) eluent temperature, (J) elution volume, (K) elution flow rate, (L) concentration of NaCl, and (M) interference ions. In (A)–(M), the concentrations of these species were 1.0 μg $L^{-1}$. All data have been normalized to the respective maxima of these species for each parameter. The error bars represent standard deviations ($n$ = 6).

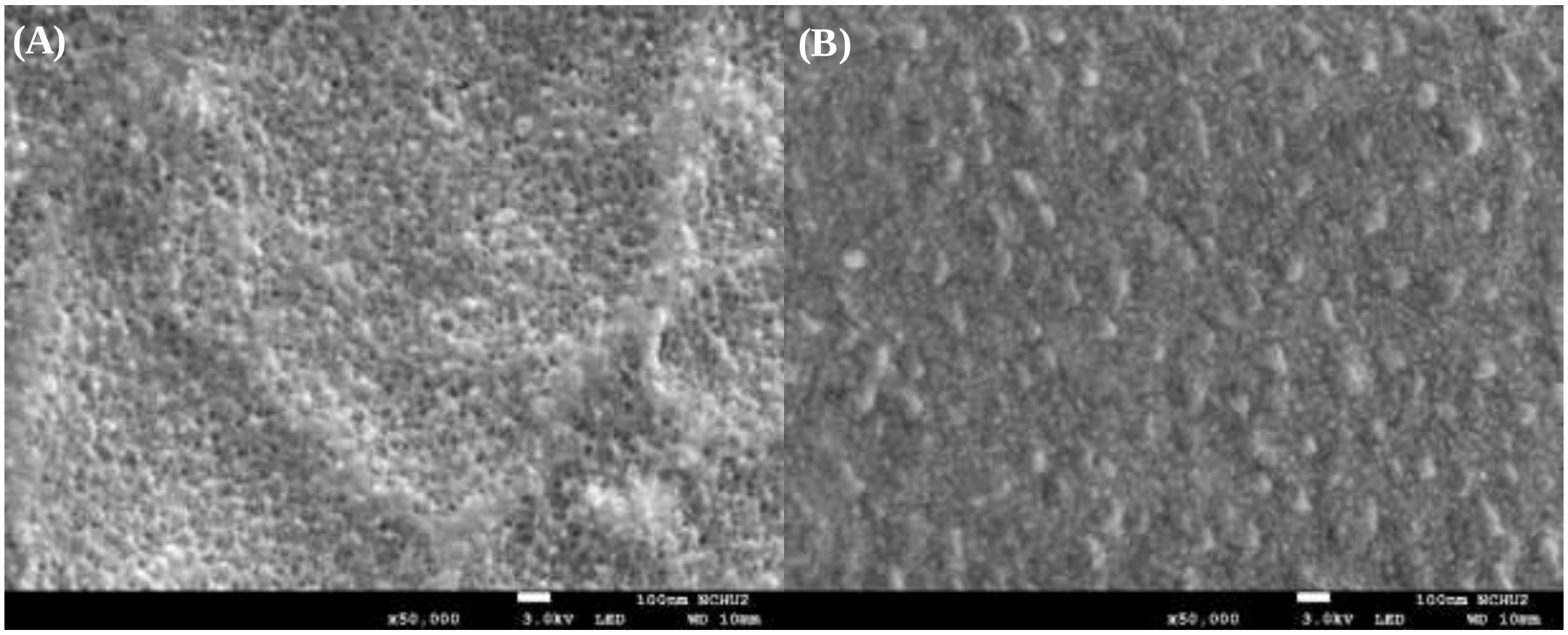


**Figure S10.** SEM images of the surfaces of the (A) nonresponsive part (1.0 °C) and (B) graphene-incorporated part (40 °C), recorded using a JEOL JSM-7800F scanning electron microscope.

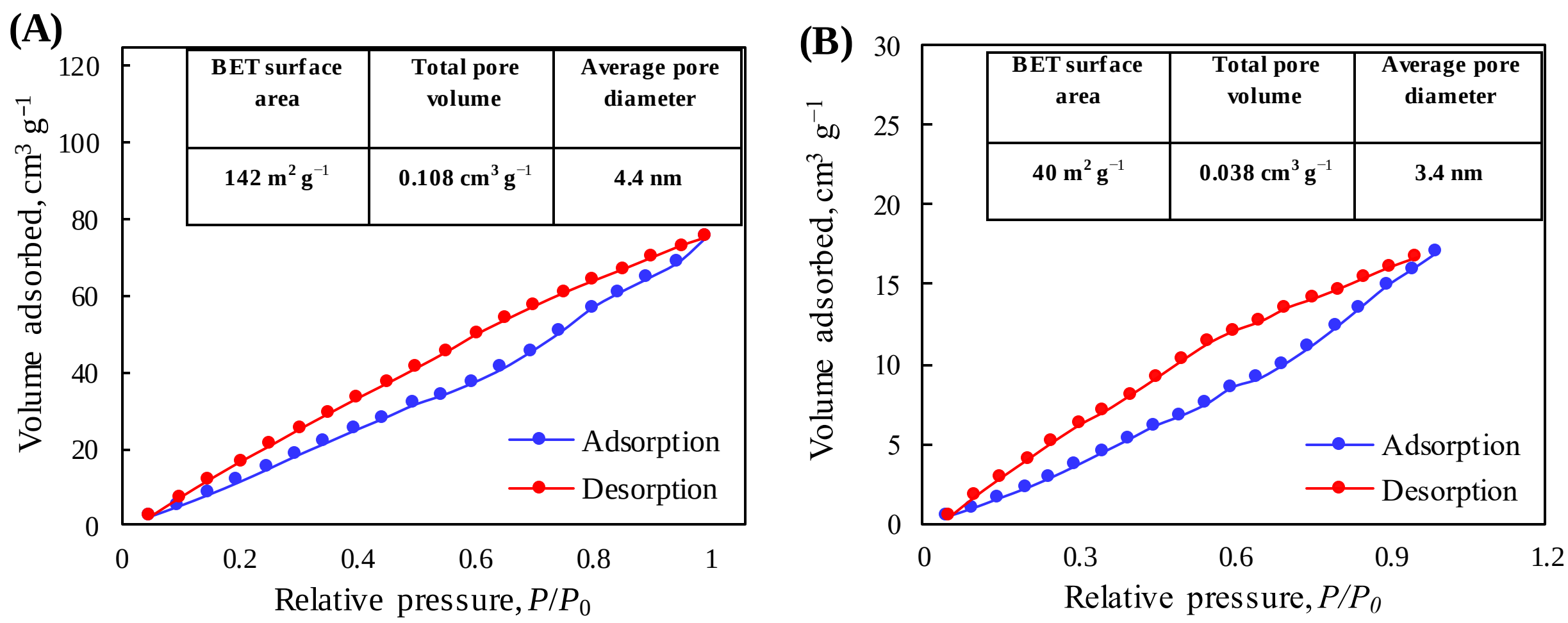


**Figure S11.** $N_2$ adsorption/desorption isotherms of the (A) nonresponsive part (1.0 °C) and (B) graphene-incorporated part (40 °C).

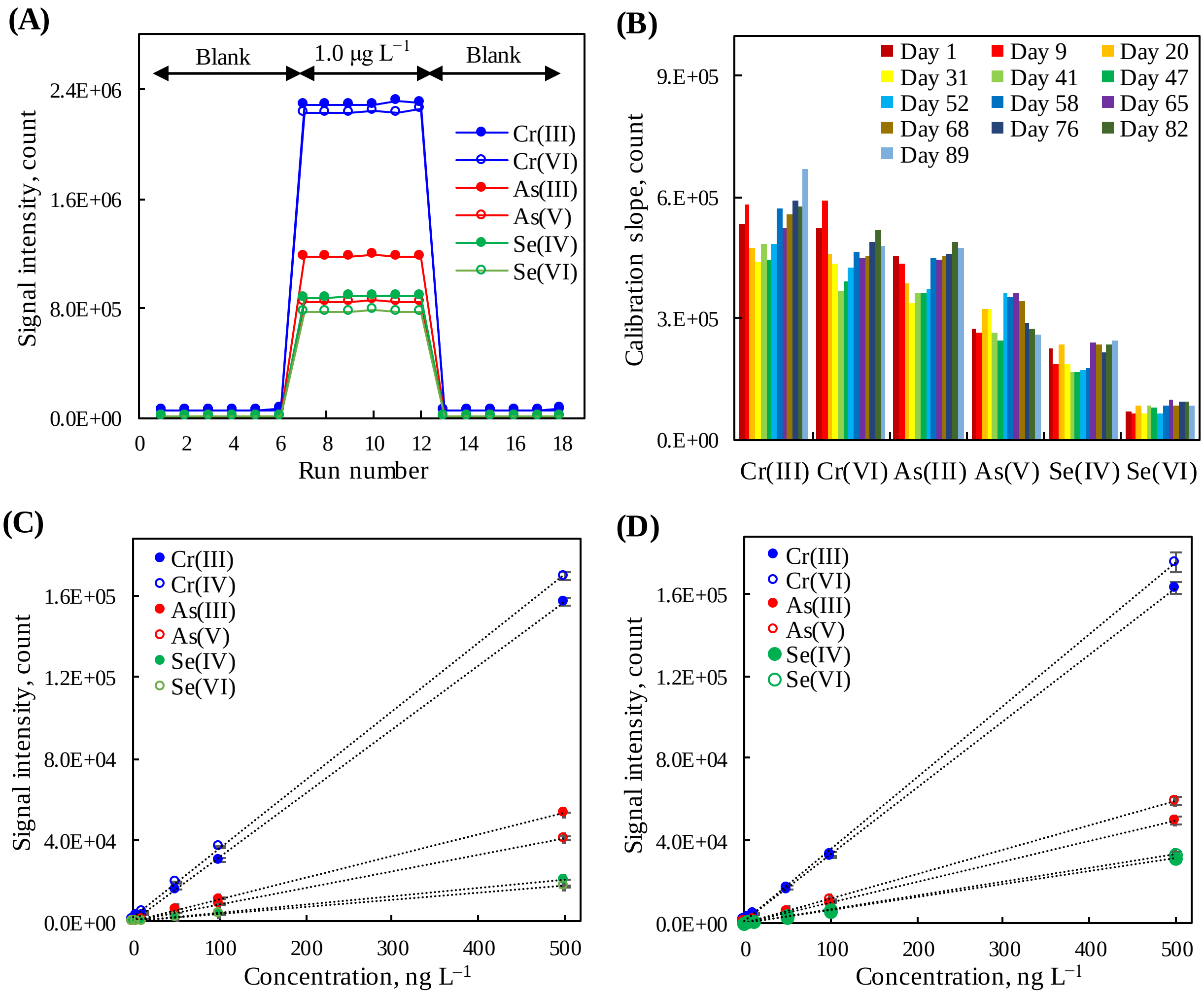


**Figure S12.** (A) Carry-over effects of these species in this automated speciation system. (B) Daily calibration slopes of these species for the same column used up to 89 days. (C) Calibration plots of these species in the automated speciation system using one SPE column with the 4D-printed NIR-actuated temperature-switchable monolithic packing. (D) Calibration plots of these species in the automated speciation system using two SPE columns with the polyNIPAM monolithic packing. In (B), the RSDs of the calibration slopes for these species were 12.5% for Cr(III), 12.5% for Cr(VI), 12.1% for As(III), 14.4% for As(V), 15.3% for Se(IV), and 14.5% for Se(VI). In (C) and (D), the error bars represent standard deviations ($n$ = 6).

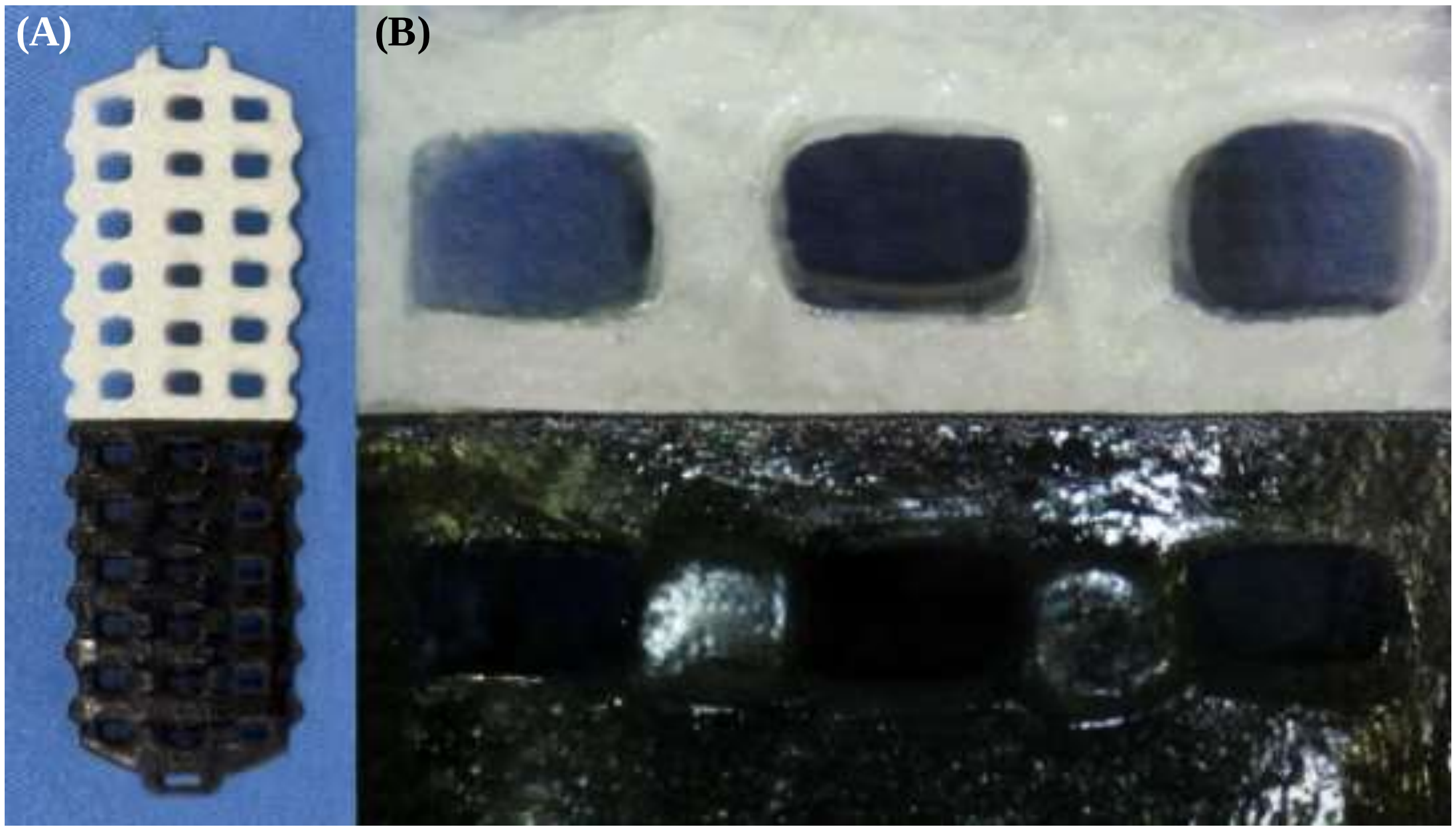


**Figure S13.** Photographs of the (A) 4D-printed temperature-switchable monolithic packing and (B) interfacial region between the nonresponsive part and the graphene-incorporated part after repeated NIR-actuated deswelling–swelling cycles.

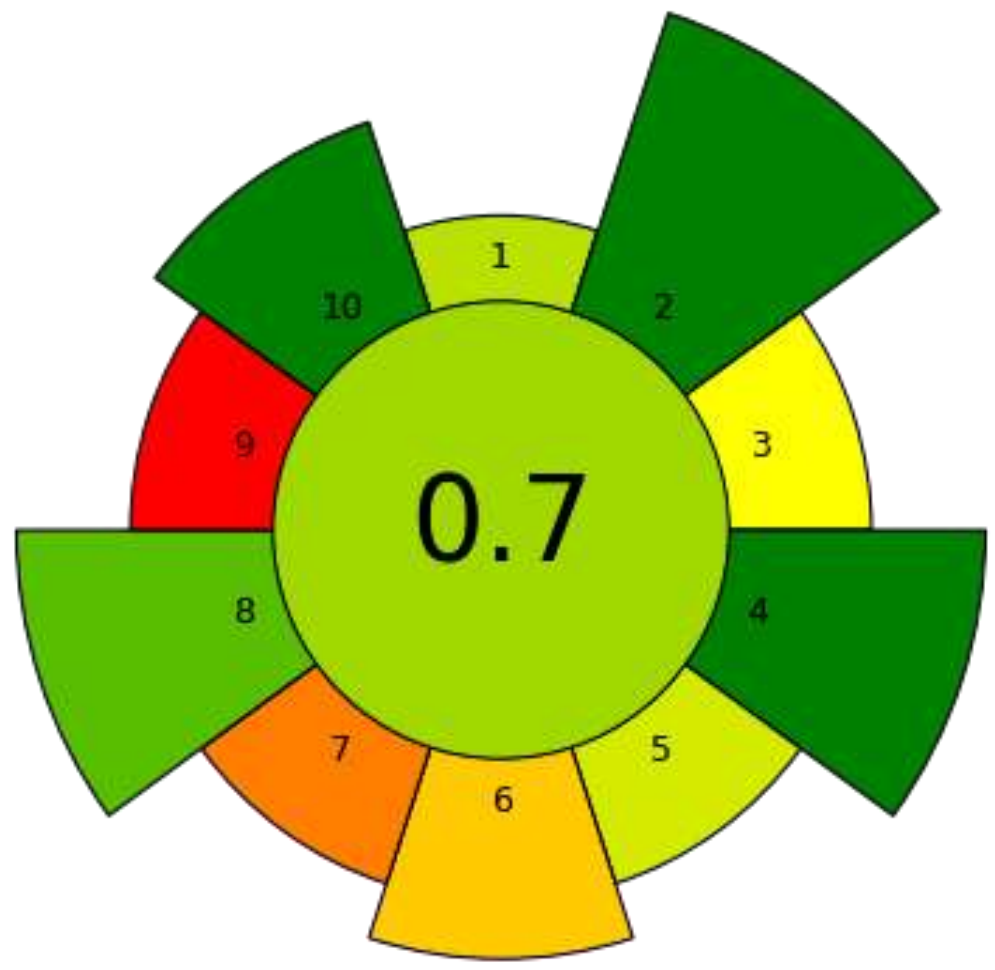


**Figure S14.** AGREEprep pictogram displaying the overall greenness score of the automated speciation system using the SPE column with the 4D-printed NIR-actuated temperature-switchable monolithic packing.

**Table S1.** Operating sequence of the automated speciation system using the SPE column with the 4D-printed NIR-actuated temperature-switchable monolithic packing

| Step | Valve position (1 → 3) | Time interval | Function |
|---|---|---|---|
| 1 | BAA | 0:00–1:30 | Sample loading/with NIR |
| 2 | AAA | 1:30–3:30 | Rinsing the column with pure water |
| 3 | AAB | 3:30–8:50 | Stopped-flow for swelling of the graphene-incorporated part and desorption of Cr(VI), As(V), and Se(VI) |
| 4 | ABB | 8:50–10:20 | Elution of Cr(VI), As(V), and Se(VI) with pure water |
| 5A | ABA | 10:20–10:58 | Elution of Cr(III), As(III), and Se(IV) with 0.5% $HNO_3$ |
| 5B | ABA | 10:58–11:50 | Elution of Cr(III), As(III), and Se(IV) with 0.5% $HNO_3$/with NIR |
| 6 | ABB | 11:50–12:20 | Rinsing the column with pure water for next loading/with NIR |

**Table S2.** Operating sequence of the automated speciation system using two SPE columns with the polyNIPAM monolithic packing

| Step | Valve position (1 → 5) | Time interval | C1 for Cr(III), As(III), and Se(IV) | C2 for Cr(VI), As(V), and Se(VI) |
|---|---|---|---|---|
| 1 | BAABA | 0:00–1:30 | sample loading | eluted with 5.0% $HNO_3$ |
| 2 | AAABA | 1:30–3:00 | rinsed by pure water | eluted with 5.0% $HNO_3$ |
| 3 | AAABB | 3:00–4:00 | rinsed by pure water | removal of residual eluent |
| 4 | ABBAA | 4:00–5:30 | eluted with 5.0% $HNO_3$ | sample loading |
| 5 | AABAA | 5:30–7:00 | eluted with 5.0% $HNO_3$ | rinsed by pure water |
| 6 | AABAB | 7:00–8:00 | removal of residual eluent | rinsed by pure water |

**Table S3.** Optimized conditions for the automated speciation system using the SPE column with the 4D-printed NIR-actuated temperature-switchable monolithic packing

| **4D-printed NIR-actuated temperature-switchable monolithic packing** | |
|---|---|
| Dimension of cuboids | 8.2 mm (L) × 0.7 mm (W) × 0.7 mm (H) |
| Interstitial spaces, mm | 1.0 mm |
| Number of cuboids per layer | 4 |
| Layers of nonresponsive part | 14 |
| Layers of NIR-actuated part | 15 |
| Concentration of NIPAM/MBA | 20%/4.0% |
| Concentration of graphene | 0.4% |
| Laser power and spot size | 6.0 W/1.0 mm |
| **Speciation using one SPE column with the 4D-printed NIR-actuated temperature-switchable monolithic packing** | |
| Sample condition | 1.0 °C; pH 2.0 (10 mM phosphate) |
| Sample loading flow rate and volume | 1.0 mL $min^{-1}$, 1.5 mL |
| Rinsing medium | $H_2O$ (1.0 °C) |
| Rinsing flow rate and volume | 1.0 mL $min^{-1}$, 2.0 mL |
| Eluent for Cr(VI), As(V), and Se(VI) | $H_2O$ (room temperature) |
| Elution flow rate and volume | 1.0 mL $min^{-1}$, 1.5 mL |
| Eluent for Cr(III), As(III), and Se(IV) | 0.5% $HNO_3$ (room temperature) |
| Elution flow rate and volume | 1.0 mL $min^{-1}$, 1.5 mL |
| Rinsing medium | $H_2O$ (room temperature) |
| Rinsing flow rate and volume | 1.0 mL $min^{-1}$, 0.5 mL |
| **ICP-MS system** | |

| | |
|---|---|
| ICP mass spectrometer | Agilent 7700x |
| Plasma forward power | 1500 W |
| Ar gas flow rate | |
| Plasma gas | 15 L min$^{-1}$ |
| Auxiliary gas | 0.9 L min$^{-1}$ |
| Carrier gas | 0.90 L min$^{-1}$ |
| Makeup gas | 0.20 L min$^{-1}$ |
| Reaction mode | He gas, 0.50 mL min$^{-1}$ |
| Sampling cone | Pt, 1-mm orifice |
| Skimmer cone | Pt, 0.4-mm orifice |
| Analysis mode | Time-resolved analysis |
| Integration time | 50 ms |
| Isotopes monitored | $^{52}Cr$, $^{75}As$, $^{82}Se$ |

**Table S4.** Operating conditions for the automated speciation system using two SPE columns with the polyNIPAM monolithic packing

| **polyNIPAM monolithic packing** | |
|---|---|
| Dimension of cuboids | 8.2 mm (L) × 0.7 mm (W) × 0.7 mm (H) |
| Interstitial spaces, mm | 1.0 mm |
| Number of cuboids per layer | 4 |
| Number of layers of cuboids | 30 |
| Concentration of NIPAM/MBA | 20%/4.0% |

| **Speciation using two columns with the polyNIPAM monolithic packing** | |
|---|---|
| Sample condition | pH 2.0 (10 mM phosphate) |
| Sample temperature | 1.0 °C for C1<br>40 °C for C2 |
| Sample loading flow rate and volume | 1.0 mL $min^{-1}$, 1.5 mL |
| Rinsing medium | $H_2O$ (1.0 °C) for C1<br>$H_2O$ (40 °C) for C2 |
| Rinsing flow rate and volume | 1.0 mL $min^{-1}$, 2.5 mL |
| Eluent | 0.5% $HNO_3$ (1.0 °C) for C1<br>0.5% $HNO_3$ (40 °C) for C2 |
| Elution flow rate and volume | 1.0 mL $min^{-1}$, 3.0 mL |
| Rinsing medium | $H_2O$ (1.0 °C) for C1<br>$H_2O$ (40 °C) for C2 |
| Rinsing flow rate and volume | 1.0 mL $min^{-1}$, 1.0 mL |

| **ICP-MS system** | |
|---|---|
| ICP mass spectrometer | Agilent 7700x |
| Plasma forward power | 1500 W |

| | |
|---|---|
| Ar gas flow rate | |
| Plasma gas | 15 L min$^{-1}$ |
| Auxiliary gas | 0.9 L min$^{-1}$ |
| Carrier gas | 0.89 L min$^{-1}$ |
| Makeup gas | 0.21 L min$^{-1}$ |
| Reaction mode | He gas, 0.50 mL min$^{-1}$ |
| Sampling cone | Pt, 1-mm orifice |
| Skimmer cone | Pt, 0.4-mm orifice |
| Analysis mode | Time-resolved analysis |
| Integration time | 50 ms |
| Isotopes monitored | $^{52}$Cr, $^{75}$As, $^{82}$Se |

**Table S5.** Analytical characteristics of the automated speciation system using two SPE columns with the polyNIPAM monolithic packing

| Element | Working range, ng $L^{-1}$ | Calibration curve | $R$ | MDL, ng $L^{-1}$ | Extraction efficiency, % | EF[b] |
|---|---|---|---|---|---|---|
| Cr(III) | 5–500 | $y$ = 325[Cr(III)][a] + 465 | 0.9999 | 0.5 | 98.9 | 3.2 |
| Cr(VI) | 5–500 | $y$ = 349[Cr(VI)][a] + 380 | 0.9998 | 0.4 | 97.2 | 2.3 |
| As(III) | 5–500 | $y$ = 99[As(III)][a] - 9 | 0.9998 | 0.7 | 92.6 | 5.4 |
| As(V) | 5–500 | $y$ = 118[As(V)][a] - 16 | 0.9995 | 0.8 | 88.0 | 4.5 |
| Se(IV) | 5–500 | $y$ = 62[Se(IV)][a] + 29 | 0.9997 | 0.7 | 59.1 | 4.3 |
| Se(VI) | 5–500 | $y$ = 66[Se(VI)][a] + 221 | 1.0000 | 0.7 | 68.8 | 3.9 |

[a]: ng $L^{-1}$.

[b]: Ratio of the elution peak heights after and before extraction (1.0 μg $L^{-1}$).

**Table S6.** Analytical characteristics of reported SPE-based elemental speciation methods for inorganic Cr, As, and Se

| Analytical scheme and detection method | Species | Sample volume, mL | Throughput, $h^{-1}$ | MDL, ng $L^{-1}$ | Extraction efficiency, % | Capacity, μg $cm^{-2}$ | Reference |
|---|---|---|---|---|---|---|---|
| one column with 4D-printed temperature-switchable monolithic packing with *in situ* switching of the hydrophobicity for extraction of all species/ICP-MS | Cr(III), Cr(VI), As(III), As(V), Se(IV), Se(VI) | 1.5 | 4.9 | 0.1–0.6 | 87.3–98.8 | 75.3–134.0 | this study |
| two columns with $TiO_2$ nanoparticle–coated porous monolith at two extraction conditions/ICP-MS | Cr(III), Cr(VI), As(III), As(V), Se(IV), Se(VI) | 2.0 | 15 | 0.7–32.3 | 84.5–98.3 | 45.4–75.9 | 17 |
| two columns respectively packed with silica-based adsorbent immobilized with poly(4,9-dioxododecane-1,12-guanidine) and silicas modified with mercaptophenyl groups at two extraction conditions/ICP-MS | Se(IV), Se(VI) | 200 | – | 0.75–1.25 | 55–99 | 2.2–2.8[a] | 16 |
| two $TiO_2$ nanoparticle–incorporated minicolumns at two extraction conditions/ICP-MS | As(III), As(V), Se(IV), Se(VI) | 0.5 | 10 | 4–128 | 14–62 | 0.40–0.76 | 14 |
| one carboxyl-functionalized organic/inorganic hybrid monolithic column for extraction of Cr(III), with calculation of Cr(VI) from total Cr/ICP-MS | Cr(III), Cr(VI) | 4 | – | 4 | – | 2.80 | 72 |

| | | | | | | | |
|---|---|---|---|---|---|---|---|
| two columns respectively packed with metal-organic framework composite nanoparticles and mercapto-functionalized metal-organic framework composite nanoparticles at two extraction conditions/ICP-MS | As(III), As(V) | 0.25 | 7 | 4.8–7.1 | – | 3.5–4.2[a] | 73 |
| two organic–inorganic hybrid monolithic capillary columns for respective extraction of As(V) and that from online oxidation of As(III) at one extraction condition/ICP-MS | As(III), As(V) | 1.0 | 5.6 | 5 | 95 | – | 20 |
| two amine- and carboxyl-bifunctionalized hybrid monolithic columns at two extraction conditions/ICP-MS | Cr(III), Cr(VI) | 3 | – | 7 | – | 1.83–2.51 | 74 |
| three minicolumns packed respectively with chelating resins and anionic ion-exchange resins at one extraction condition/ICP-MS | Cr(III), Cr(VI) | 4.0 | 7.5 | 9–30 | – | – | 13 |
| one microcolumn packed with functionalized multiwalled carbon nanotubes for extraction of high-valent species, with low-valent species calculated from total concentrations/ICP-MS | Cr(III), Cr(VI), As(III), As(V), Se(IV), Se(VI) | 4 | – | 15–38 | – | 7.02–9.79[a] | 71 |
| one column packed with nanometer-sized $Al_2O_3$ for extraction of Se(IV) and Se(VI), and another column | Se(IV), Se(VI) | 4 | 7.4 | 50–120 | – | 3.8–5.1[a] | 70 |

| | | | | | | | |
|---|---|---|---|---|---|---|---|
| packed with dimercaptosuccinic acid-modified mesoporous $TiO_2$ for extraction of Se(IV)/ICP-MS | | | | | | | |
| one column packed with anionic ion-exchange resins for extraction of Cr(VI) and that from oxidation of Cr(III) at one extraction condition/FAAS | Cr(III), Cr(VI) | 150 | – | 7700–8600 | > 88 | – | 18 |

[a]: mg $g^{-1}$

**Table S7.** Analytical results of reference materials ($n$ = 6)

| | | CASS-6 | SLRS-5 | 1643f | Trace Elements Urine L-2 |
|---|---|---|---|---|---|
| Cr | Certified value, μg $L^{-1}$ | 0.100 ± 0.016 | 0.208 ± 0.023 | 18.50 ± 0.10 | 21.2 ± 4.3 |
| | Measured Cr(III), μg $L^{-1}$ | 0.10 ± $0.00_2$ | 0.21 ± 0.01 | 18.50 ± 0.30 | 21.36 ± 0.16 |
| | Measured Cr(VI), μg $L^{-1}$ | ND | ND | ND | ND |
| | Measured $Cr_{total}$, μg $L^{-1}$ | 0.01 ± $0.00_2$ | 0.21 ± 0.01 | 18.50 ± 0.30 | 21.36 ± 0.16 |
| | Relative error, % | +2.0 | +0.6 | 0 | +0.7 |
| | $p$ value | 1.0000 | 0.8629 | 1.0000 | 0.9358 |
| | Cr(III) after spiking, μg $L^{-1}$ | 0.36 ± 0.01[a] | 0.46 ± 0.01[a] | 43.63 ± 0.79[b] | 46.78 ± 1.12[b] |
| | Cr(VI) after spiking, μg $L^{-1}$ | 0.24 ± $0.00_4$[a] | 0.25 ± $0.00_2$[a] | 23.54 ± 0.67[b] | 24.90 ± 0.76[b] |
| | Spike recovery of Cr(III), % | 102 | 105 | 101 | 108 |
| | Spike recovery of Cr(VI), % | 94 | 94 | 94 | 94 |
| As | Certified value, μg $L^{-1}$ | 0.50[c] | 0.413 ± 0.039 | 57.42 ± 0.38 | 184 ± 37 |
| | Measured As(III), μg $L^{-1}$ | 0.51 ± 0.01 | 0.41 ± $0.00_4$ | 36.48 ± 0.23 | 146.21 ± 1.94 |
| | Measured As(V), μg $L^{-1}$ | ND | ND | 20.89 ± 0.32 | 36.96 ± 0.25 |
| | Measured $As_{total}$, μg $L^{-1}$ | 0.51 ± 0.01 | 0.41 ± $0.00_4$ | 57.37 ± 0.40 | 182.17 ± 1.95 |
| | Relative error, % | +2.8 | –0.8 | –0.1 | –1.0 |
| | $p$ value | – | 0.8684 | 0.8445 | 0.9148 |
| | As(III) after spiking, μg $L^{-1}$ | 0.77 ± 0.01[a] | 0.65 ± $0.00_4$[a] | 62.69 ± 0.83[b] | 170.82 ± 2.06[b] |
| | As(V) after spiking, μg $L^{-1}$ | 0.24 ± $0.00_2$[a] | 0.25 ± $0.00_4$[a] | 44.68 ± 0.55[b] | 61.11 ± 1.15[b] |
| | Spike recovery of As(III), % | 100 | 103 | 105 | 102 |
| | Spike recovery of As(V), % | 97 | 96 | 95 | 95 |
| Se | Certified value, μg $L^{-1}$ | – | – | 11.700 ± 0.081 | 70.1 ± 14.1 |
| | Measured Se(IV), μg $L^{-1}$ | 0.31 ± 0.01 | 0.16 ± $0.00_5$ | 11.76 ± 0.33 | 70.99 ± 0.89 |
| | Measured Se(VI), μg $L^{-1}$ | ND | ND | ND | ND |

| | | | | |
|---|---|---|---|---|
| Measured $Se_{total}$, μg $L^{-1}$ | 0.31 ± 0.01 | 0.16 ± $0.00_5$ | 11.76 ± 0.33 | 70.99 ± 0.89 |
| Relative error, % | – | – | +0.5 | +1.3 |
| *p* value | – | – | 0.7033 | 0.8915 |
| Se(IV) after spiking, μg $L^{-1}$ | 0.57 ± 0.01[a] | 0.41 ± 0.01[a] | 37.83 ± 1.07[b] | 95.50 ± 2.03[b] |
| Se(VI) after spiking, μg $L^{-1}$ | 0.24 ± 0.01[a] | 0.25 ± 0.01[a] | 24.76 ± 1.04[b] | 24.85 ± 0.68[b] |
| Spike recovery of Se(IV), % | 104 | 103 | 104 | 101 |
| Spike recovery of Se(VI), % | 97 | 98 | 99 | 92 |

[a]: Spiked concentration: 0.25 μg $L^{-1}$.

[b]: Spiked concentration: 25 μg $L^{-1}$.

[c]: Information value.

**Table S8.** Analytical results of environmental water and human urine samples ($n$ = 6)

| | | Seawater | River water | Ground water | Agricultural wastewater | Urine |
|---|---|---|---|---|---|---|
| Cr | Measured Cr(III), μg $L^{-1}$ | 0.14 ± $0.00_4$ | 0.08 ± $0.00_3$ | 0.10 ± $0.00_3$ | 1.42 ± 0.04 | 0.76 ± 0.02 |
| | Measured Cr(VI), μg $L^{-1}$ | ND | ND | ND | ND | ND |
| | Measured $Cr_{total}$, μg $L^{-1}$ | 0.14 ± $0.00_4$ | 0.08 ± $0.00_3$ | 0.10 ± $0.00_3$ | 1.42 ± 0.04 | 0.76 ± 0.02 |
| | Cr(III) after spiking,[a] μg $L^{-1}$ | 0.39 ± 0.01 | 0.34 ± 0.01 | 0.35 ± 0.01 | 1.67 ± 0.03 | 1.01 ± 0.03 |
| | Cr(VI) after spiking,[a] μg $L^{-1}$ | 0.25 ± 0.01 | 0.25 ± $0.00_2$ | 0.25 ± $0.00_5$ | 0.26 ± 0.01 | 0.25 ± 0.01 |
| | Spike recovery of Cr(III), % | 100 | 102 | 100 | 102 | 101 |
| | Spike recovery of Cr(VI), % | 101 | 99 | 102 | 103 | 100 |
| As | Measured As(III), μg $L^{-1}$ | 0.56 ± $0.00_5$ | 0.08 ± $0.00_1$ | 0.07 ± $0.00_2$ | 2.00 ± 0.03 | 0.20 ± 0.01 |
| | Measured As(V), μg $L^{-1}$ | 0.10 ± $0.00_1$ | ND | ND | 0.15 ± $0.00_4$ | 0.08 ± $0.00_1$ |
| | Measured $As_{total}$, μg $L^{-1}$ | 0.66 ± $0.00_5$ | 0.08 ± $0.00_1$ | 0.07 ± $0.00_2$ | 2.16 ± 0.03 | 0.28 ± 0.01 |
| | As(III) after spiking,[a] μg $L^{-1}$ | 0.82 ± 0.01 | 0.33 ± $0.00_2$ | 0.32 ± $0.00_4$ | 2.26 ± 0.03 | 0.45 ± $0.00_3$ |
| | As(V) after spiking,[a] μg $L^{-1}$ | 0.35 ± 0.01 | 0.26 ± $0.00_4$ | 0.25 ± $0.00_3$ | 0.41 ± $0.00_5$ | 0.33 ± $0.00_4$ |
| | Spike recovery of As(III), % | 102 | 101 | 100 | 102 | 101 |
| | Spike recovery of As(V), % | 100 | 102 | 98 | 101 | 98 |
| Se | Measured Se(IV), μg $L^{-1}$ | 1.50 ± 0.04 | 1.58 ± 0.03 | 0.15 ± $0.00_3$ | 1.61 ± 0.03 | 1.45 ± 0.02 |
| | Measured Se(VI), μg $L^{-1}$ | ND | ND | ND | 1.86 ± 0.04 | ND |
| | Measured $Se_{total}$, μg $L^{-1}$ | 1.50 ± 0.04 | 1.58 ± 0.03 | 0.15 ± $0.00_3$ | 3.46 ± 0.05 | 1.45 ± 0.02 |
| | Se(IV) after spiking,[a] μg $L^{-1}$ | 1.75 ± 0.04 | 1.83 ± 0.06 | 0.40 ± 0.01 | 1.86 ± 0.05 | 1.69 ± 0.05 |
| | Se(VI) after spiking,[a] μg $L^{-1}$ | 0.25 ± 0.01 | 0.26 ± 0.01 | 0.24 ± 0.01 | 2.11 ± 0.09 | 0.25 ± 0.01 |
| | Spike recovery of Se(IV), % | 101 | 102 | 101 | 99 | 99 |
| | Spike recovery of Se(VI), % | 99 | 103 | 98 | 102 | 102 |

[a]: Spiked concentration: 0.25 μg $L^{-1}$.
